# Mechanistic Insights into the Binding of Class IIa HDAC Inhibitors toward Spinocerebellar Ataxia Type-2: A 3D-QSAR and Pharmacophore Modeling Approach

**DOI:** 10.3389/fnins.2016.00606

**Published:** 2017-01-10

**Authors:** Siddharth Sinha, Sukriti Goyal, Pallavi Somvanshi, Abhinav Grover

**Affiliations:** ^1^Department of Biotechnology, TERI UniversityNew Delhi, India; ^2^Department of Bioscience and Biotechnology, Banasthali UniversityTonk, India; ^3^School of Biotechnology, Jawaharlal Nehru UniversityNew Delhi, India

**Keywords:** spinocerebellar ataxia type-2, polyglutamine disorder, HDAC inhibitors, 3D-QSAR, pharmacophore modeling, ROC curve, enrichment factor (EF), goodness of hit (GH)

## Abstract

Spinocerebellar ataxia (SCA-2) type-2 is a rare neurological disorder among the nine polyglutamine disorders, mainly caused by polyQ (CAG) trinucleotide repeats expansion within gene coding ataxin-2 protein. The expanded trinucleotide repeats within the ataxin-2 protein sequesters transcriptional cofactors i.e., CREB-binding protein (CBP), Ataxin-2 binding protein 1 (A2BP1) leading to a state of hypo-acetylation and transcriptional repression. Histone de-acetylases inhibitors (HDACi) have been reported to restore transcriptional balance through inhibition of class IIa HDAC's, that leads to an increased acetylation and transcription as demonstrated through *in-vivo* studies on mouse models of Huntington's. In this study, 61 di-aryl cyclo-propanehydroxamic acid derivatives were used for developing three dimensional (3D) QSAR and pharmacophore models. These models were then employed for screening and selection of anti-ataxia compounds. The chosen QSAR model was observed to be statistically robust with correlation coefficient (*r*^2^) value of 0.6774, cross-validated correlation coefficient (*q*^2^) of 0.6157 and co-relation coefficient for external test set (*pred*_*r*^2^) of 0.7570. A high *F*-test value of 77.7093 signified the robustness of the model. Two potential drug leads ZINC 00608101 (SEI) and ZINC 00329110 (ACI) were selected after a coalesce procedure of pharmacophore based screening using the pharmacophore model ADDRR.20 and structural analysis using molecular docking and dynamics simulations. The pharmacophore and the 3D-QSAR model generated were further validated for their screening and prediction ability using the enrichment factor (EF), goodness of hit (GH), and receiver operating characteristics (ROC) curve analysis. The compounds SEI and ACI exhibited a docking score of −10.097 and −9.182 kcal/mol, respectively. An evaluation of binding conformation of ligand-bound protein complexes was performed with MD simulations for a time period of 30 ns along with free energy binding calculations using the g_mmpbsa technique. Prediction of inhibitory activities of the two lead compounds SEI (7.53) and ACI (6.84) using the 3D-QSAR model reaffirmed their inhibitory characteristics as potential anti-ataxia compounds.

## Introduction

Ataxia is a term indicating the lack of coordination and movement as a result of degeneration of cerebellum, the coordination center of brain. Molecular pathology behind spinocerebellar ataxia type-2 (SCA-2) is the expansion of cytosine-adenine-guanine (CAG) repeat. Normal allele of SCA2 comprises of 13–37 repeats of CAG whereas the mutant allele comprises of 38–53 repeats (Armstrong et al., 2005). SCA2 is characterized through progressive gait and limb in-coordination, muscle weakness, slurring of speech, decreased vibration sense, and dysarthria. The onset of symptoms occurs in mid-40s and with the progression of diseased state, the patient is confined to wheel chair within a time span of 5–7 years. Till date there is no treatment available for SCA-2. Recent studies on SCA-2 have reported that the mutant protein having expanded trinucleotide (CAG) repeat sequesters the transcriptional factors affecting the expression of gene through transcriptional de-regulation (Brusco et al., 2004).

Expression of gene is regulated through alteration in chromatin. Chromatin has a high level complex structure that arises from the assembly of nucleosomes, an octamer of histone proteins viz., H2A, H2B, H3, and H4 enclosing 150 base pair of DNA. Regulation of gene transcription is controlled by interaction between the Histone and DNA. Chemical modifications particularly the N-e-acetylation of lysine residues is found in histone proteins (Bassett and Barnett, 2014). Acetylation is a widely investigated translational modification in comparison to other modifications which include phosphorylation and methylation. It involves the transfer of –COCH_3_ group from acetyl CoA to lysine side chain and is revamped through Histone acetyl transferases (HAT) and Histone de-acetylase (HDAC's). HAT functions by promoting acetylation of histone proteins thereby increasing gene transcription while HDAC's remove the acetyl group from the histone resulting in the repression of gene transcription. In disease state, the expanded polyQ repeats within the mutant ataxin-2 protein sequesters transcriptional cofactors i.e., CREB, A2BP1, TDP43 resulting in the state of hypo-acetylation. Excessive de-acetylation of the histone proteins has been associated to polyglutamine disorders esp. Spinocerebellar ataxia's and Huntington's. Although the definite molecular pathology for de-regulation has not been fully decoded till date, it has been well-documented that transcriptional repression due to hypo-acetylation is one of the main reasons for spinocerebellar ataxia (Butler and Bates, 2006).

HDAC's superfamily consists of 18 sub-types that have been branched in four classes (Class I–IV), classified in accordance with phylogenetic comity, catalytic mechanism, expression pattern and similarity with yeast de-acetylase (Bertrand, 2010). HDAC's are structurally and functionally distinct as class I, II, and IV are termed classical HDAC's (cofactor is Zn^2+^) while class III are homologs of the yeast silent information regulator2 (*SIR2*), an NAD^+^ dependent histone de-acetylase. Class II HDAC's are sub-classified into class IIa and class IIb consisting of HDAC 4, 5, 7, and 9, and HDAC 6 and 10, respectively (Bottomley et al., 2008). Class IV consists of single member HDAC 11. Class III HDAC's, structurally distinct from classical HDAC's, comprises of Silent-Information-Regulator (SIRT's 1–7). Class IIa HDAC demonstrates tissue specific presence in brain indicating their significance toward gene transcription in neurons through transcription cofactors in comparison to class I HDAC's that are expressed globally. Present study focuses on Class IIa HDAC's as they have nuclear localization signal (NLS) and nuclear export signal (NES) which is a prerequisite for the histone de-acetylation. This leads to phosphorylation of the serine residue resulting in the export of HDAC's from nucleus into the cytoplasm where it binds to protein 14-3-3 and then shuttle back to nucleus with the loss of phosphorylation and dissociation with protein 14-3-3 (Didonna and Opal, 2015). HDAC's active site comprises of two domains, a C terminal catalytic domain and an N terminal regulatory domain (Di Giorgio et al., 2015). These domains are involved in charge relay system through removal of acetyl group, and surrounds the Zn^2+^ ion having two histidine aspartic dyads [His802-Asp838 and His803-Asp845].

HDAC inhibitors (HDACi) comprise of diverse array of natural and synthetic compounds, broadly classified into four classes in accordance with their potency, namely, Hydroxamic acids, Cyclic peptides, Benzamides and Aliphatic acids (Juvale et al., 2006). HDACi functions by binding to Zn^2+^ ion thereby dysfunctioning the charge relay system involved in the de-acetylation of lysine residue. Basic structure of HDACi comprises of a metal binding group, a hydrophobic cap and an aliphatic chain which links the hydrophobic cap to the metal binding group. Variation in the length of aliphatic chain is known to have consequent effect on inhibitory potential of the compounds. This study focuses on the hydroxamate moiety based HDACi and their class specificity toward class IIa HDAC i.e., HDAC4. HDACi such as suberoylanilidehydroxamic acid (SAHA), TSA, FSK-228, Phenyl butyrate, and Valproic acid (VPA) have been reported under clinical trials, many of which do not demonstrate class specific selectivity. TSA has been reported as pan-HDAC inhibitor whereas inhibitors like Valproic acid, FK-228, SAHA, exhibit class II specific inhibition. HDACi possessing hydroxamte moiety have been widely investigated toward various polyglutamine disorders (Price et al., 2007). *In-vivo* studies have shown that SAHA and Phenyl butyrate improves the motor deficit in R6/2 and N171-82Q transgenic mouse model of Huntington's respectively (Voet and Zhang, 2012). Structural studies have also revealed the binding association of HDACi like TSA and SAHA with histone de-acetylase protein through interning its aliphatic chains and co-ordinating with the Zn^2+^ ion (Hockly et al., 2003).

In this study, we selected a congeneric series of 61 hydroxamic acid derivatives exhibiting histone de-acetylase inhibitory properties toward spinocerebellarataxia type-2; which has not been reported till date to the best of our knowledge. In order to search for novel compounds possessing anti-HDAC therapeutic properties, we selected 1,2 di-arylcyclo-propanehydroxamic acid derivatives for 3D-QSAR studies that co-relates the biological and physiochemical properties of the compounds against HDAC4. A combined screening methodology involving pharmacophore screening along with prediction of inhibitory potential of screened compounds using 3D-QSAR was adopted. The potential lead compounds were validated through an extensive structural analysis performed with molecular docking and dynamics simulations study. Present study provides valuable insight toward the role of di-aryl cyclo-propane hydroxamic acids as an ataxia agents and evaluation of lead compound identified through pharmacophore modeling and 3D-QSAR model.

## Materials and methods

### Protein selection and preparation

HDAC's superfamily has been classified into four groups consisting of 18 members on the basis of phylogeny and sequence homology. Class IIa HDAC4 protein (PDB ID: 4CBY) was selected owing to its various novel features. Firstly, they possess a N and a C terminal region comprising of glutamine rich domain and catalytic de-acetylase domain, known to be involved in various signaling pathway through specific post translational modifications including nuclear and cytoplasmic shuttling. This domain also consists of catalytic domain in a “closed-loop” form, reported necessary for the enzymatic activity (Bürli et al., 2013). The second novel feature of class IIa HDAC is that it possesses a bigger active site in comparison to class I HDAC, due to mutation of a tyrosine into histidine, Y967H in HDAC4 (Bottomley et al., 2008). The selected HDAC4 structure was prepared using the protein preparation wizard in the Schrodinger package. The protein was optimized using the OPLS all atom force field using gromacs version 4.6.5.

### Hydroxamate dataset for 3D-QSAR and pharmacophore modeling

A series of 61 di-arylcyclo-propanehydroxamicacid derivatives with inhibitory properties against histone de-acetylase (HDAC's) were selected for 3D-QSAR model-generation (Bürli et al., 2013). The alignment of compounds with a common template resulted in a total of 44 compounds with lower RMSD-values (Schreiber and Keating, 2011). Compounds possessing higher RMSD form alternative modes of binding in comparison to the one having lower RMSD. Compounds exhibiting lower RMSD have similar orientation as the crystallographic structure indicating optimal alignment (Kundrotas and Vakser, 2013). 2D structures of the template (a common substructure of the congeneric series) along with the other hydroxamic derivatives were drawn using the Marvin Sketch (MarvinSketch)^1^. VLife Sciences Software (MDS)^2^ was used for converting 2D structures into 3D (Goyal S. et al., 2014). The structures were analyzed utilizing force field batch minimization using selected default parameters for the model generation except the final equation consisting of four descriptors and value of 1.0 as variance cut-off.

### Force field computation

The biological activity of 44 di-aryl cyclo-propanehydroxamic acid derivatives were input in form of negative logarithm of IC_50_ i.e., pIC_50_ for force field calculations. Force field computation was carried out having default grid dimensions including steric, electrostatic and hydrophobic descriptors while with dielectric constant as 1.0. Gasteiger-Marsili was chosen as charge type for computation (Kumar et al., 2016). Out of 7148 descriptors calculated, only 1233 were selected after eliminating the static rank. Static properties are statistically similar for each point thus evidently not involved in affecting the inhibitory property of the compounds. Hence, these invariable descriptors were eliminated during QSAR model generation (Goyal M. et al., 2014).

### 3D-QSAR model generation

In this study, we selected molecular field analysis along with PLS regression technique for generating 3D-QSAR model. Molecular properties such as electrostatic and steric descriptors were selected as independent variable whereas the activity pIC_50_ as dependent variable. Statistical external validation was performed in which the dataset was classified into the test and training set. Eighty-five percent of the dataset molecules were classified as training set using random selection procedure, in order to accomplish the diversity of the training set for the whole descriptor space of the overall dataset (Martin et al., 2012). This was achieved using the following criteria: firstly, the training set molecules were structurally diverse enough to cover the whole descriptor space (Ajay and Bedadurge, 2013) and secondly, the representative points in the training and test set were close to each other (Jain et al., 2011).

### Validation of 3D-QSAR model

The predictive power of the QSAR model generated is defined as the ability to predict the biological activity of the molecules that were not used for model generation. Thus, a QSAR model is required to be checked for various quality measures before it can be employed for screening of new chemical entity. This makes validation a significant part of QSAR modeling prior to the predictions. The acceptability of a regression based QSAR model relies upon different statistical parameters such that the value of predicted activity does not differ much from the experimentally defined biological activity i.e., the net residual between the predicted and experimental activities is zero or close to zero. Validation of the selected models was ascertained by statistical parameters such as *r*^2^, *q*^2^, *pred*_*r*^2^, *Z*-score, n (no of compounds), k (variables), best_ran_*q*^2^, best_ran_*r*^2^, *F*-test (Fischer's value). Further, for a QSAR model to be statistically significant and robust it must fulfill the following criteria i.e., *q*^2^, *r*^2^ > 0.6, *pred*_*r*^2^ > 0.5 (Sinha et al., 2016) and *F*-test > 30, along with low standard error values. Validation of the QSAR model is performed using the test set, since they do not contribute to the model development and are considered as the part of external validation. On the other hand, training set forms a part of internal validation. Receiver-operating characteristics curve (ROC; Speck-Planche et al., 2012) analysis was performed for the QSAR model for validating its ability to predict the identified HDAC active compounds from a large testing list of actives and decoy (Verdonk et al., 2004; Kirchmair et al., 2008). The ROC curve demonstrates the sensitivity (Se, true positive rate) for any possible change in the number of compounds (n) as function of (1−Sp), Sp is defined as specificity or false negative rate.

$$\begin{array}{lll} \text{Sensitivity }\left(\text{Se}\right) & = & \frac{\text{Number}\;\text{of}\;\text{selected}\;\text{actives}}{\text{Total}\;\text{number}\;\text{of}\;\text{actives}}\\ \text{Se} & = & \frac{\text{TP}}{\text{TP}+\text{FN}}\\ \text{Specificity }\left(\text{Sp}\right) & = & \frac{\text{Number}\;\text{of}\;\text{discarded}\;\text{inactives}}{\text{Total}\;\text{number}\;\text{of}\;\text{inactives}}\\ \text{Sp} & = & \frac{\text{TN}}{\text{TN}+\text{FP}} \end{array}$$

Here, TP is defined as number of active compounds selected and FN is the number of active compounds discarded. Whereas, TN is the number of discarded decoys while FP is the number of selected decoys (inactive). ROC curve was plotted by setting the score of the active molecules as thresholds and consequently the number of active and decoy with the dataset was counted for calculated of senstivity and specificity. This calculation was repeated for the active molecules with second and third highest score for all the actives (**Table 3B**).

### Internal and external validation

Internal validation (*q*^2^) of the QSAR model is calculated using the given equation:

$$\begin{array}{l} q^{2}=1-\frac{\sum {\left(y_{i}-{ŷ}_{i}\right)}^{2}}{\sum {\left(y_{i}-y_{mean}\right)}^{2}} \end{array}$$

where *y*_*i*_ and ŷ_*i*_ are the actual and predicted activities of the *i*th molecule, respectively, and *y*_*mean*_ is the average activity of all molecules in the training set.

External validation (*pred*_*r*^2^) defines the co-relation between the actual and predicted activity; calculated through using the given equation:

$$\begin{array}{l} \text{pred}\_r^{2}=1-\frac{\sum {\left(y_{i}-{ŷ}_{i}\right)}^{2}}{\sum {\left(y_{i}-y_{mean}\right)}^{2}} \end{array}$$

where *y*_*i*_ and ŷ_*i*_ are the actual and predicted activities of the *i*th molecule respectively, and *y*_*mean*_ is the average activity of all the molecules in the test set.

*Y* randomization test was performed through contemplating linear model with the one derived from data set (Rucker et al., 2007), several different models were generated through rearranging the training set in order to measure them with the 3D-QSAR model on the basis of *Z*-score (Samal et al., 2013). Value of *Z*-score is given as:

$$\begin{array}{l} Z\;score=\frac{h-\mu }{\sigma } \end{array}$$

where, *h* is the *q*^2^-value calculated for the actual dataset, μ is the average *q*^2^ and σ is the standard deviation calculated for various models built on different random data sets.

### Pharmacophore-modeling

Pharmacophore hypothesis consists of aggregation of the conceivable models having steric and automated electronic features required for the atomic interactions with its target. Pharmacophore modeling was performed using PHASE 3.0 module of Schrödinger (Dixon et al., 2006) constituting of 44 hydroxamic derivatives. PHASE comprises of six pharmacophoric features which are [1] hydrogen bond acceptor, [2] hydrogen bond donor, [3] positively ionizable, [4] negatively ionizable, [5] hydrophobic group, [6] aromatic ring. The pharmacophore is created on the basis of theses identified features and is further utilized for searching anti-ataxia compounds having similar characteristics to that of the developed pharmacophore; as the calibration is placed on pharmacophoric features over the atomic interactions (Koushik Kumar et al., 2015) thereby resulting in a better representation of the binding pattern interaction of the identified compounds with the catalytic dyad of the HDAC4.

### Common pharmacophore hypothesis generation

Structures of inhibitors were pre-processed using *ligprep* (Ligprep, 2015) with OPLS_2005 force field. Pharmacophore from all conformations of the inhibitors were examined using the common pharmacophore features. Pharmacophore exhibiting identical features in terms of spatial arrangements were clustered together and were then examined by scoring procedure for ranking all the hypotheses. The pharmacophore that yielded the best alignment of the chosen inhibitors and thus possessing highest score was identified. The chosen hypothesis was then used to search for compounds in a dataset of known chemical libraries with a must match criteria of four out of five features.

### Screening of database using the selected pharmacophore hypothesis

Database prepared from a large dataset consisting of chemical compounds from FDA approved drugs, Zinc database etc., was screened for structures matching the hypothesis of the pharmacophore model. The search methodology comprised of two steps. The first step involved searching of the database for 3D arrangement of pharmacophoric sites with similar site types and intersite distances in comparison to the selected hypothesis. After finding relevant structure, its information was written to the match file. In the second fetch step, the most relevant conformers, i.e., hits, were retrieved from the database with the help of the match file and were then aligned to the hypothesis. All the hits above a fitness score of 1.0 or close to 1.0 were fetched and analyzed further (Kaur et al., 2012).

### High throughput virtual screening (HTVS) of the hits generated through *In-silico* molecular docking studies

The compounds having anti-ataxia properties identified on the basis of similarity with generated pharmacophoric hypothesis were analyzed in terms of their binding affinity and mode of interaction with HDAC4 using high throughput virtual screening (HTVS) along with extra precision (XP) protocol of molecular docking in Glide, Schrodinger (Friesner et al., 2004).

Virtual screening in recent times has emerged as one of the pioneer technique in order to identify potent leads toward the selection of potential drugs. Here the protein crystal structure (PDB ID:4CBY) obtained from protein data bank (www.rcsb.org) was docked with top scoring compounds selected on the basis of the pharmacophoric features such as survival and fitness score. The compounds were first docked with HDAC4 through high throughput virtual screening protocol (HTVS), the compounds possessing a binding energy greater than threshold value of −8.0 kcal/mol (in magnitude) were re-docked with extra precision (XP) module in order to check the veracity of the result. Protein was prepared with protein preparation wizard where the crystal water molecules and non-bonded heteroatoms were removed. It also involved hydrogen bond addition, creation of disulfide bonds and conversion of selenomethionine to methionine. The structures of potent inhibitors were prepared using the Ligprep module of Schrödinger. The post docking interaction studies for the inhibitor-enzyme complex was performed using UCSF chimera (Pettersen et al., 2004) and ligplot (Wallace et al., 1995).

### Molecular dynamics simulations of the top scoring compounds

A 20 ns long dynamic simulation was carried for the inhibitor-enzyme complex. Gromacs version 4.6.5 (Berendsen et al., 1995) was used to carry out the simulations for identifying druggable sites in the class IIa HDAC4 (PDB ID: 4CBY). PRODRG2 server (Schüttelkopf and van aalten, 2004) was utilized for creating the gromacs topology of the inhibitors for the simulation studies. The force field selected was OPLS-AA/L all-atom (van der Spoel et al., 2005; Sehrawat et al., 2015). The inhibitor-protein complex was at the center of 100 × 100 × 100 Å cubic grid, which was solvated with 10980 TIP3P water molecules and neutralized with 8 Na^+^ ions (Hess and van der Vegt, 2006). Equilibration of the inhibitor-enzyme complex along with energy minimization was carried out at constant pressure of 1 atm and temperature (NPT) of 298K with time interval of 2 femto second (fs; Tandon and Sinha, 2011). A 20 ps simulation was carried out at constant volume and temperature (NVT) with pressure 1 atm. Finally, 20 ns long molecular dynamics simulations was performed at temperature of 298K and pressure 1 atm.

### Free energy calculations through g_mmpbsa

Molecular mechanics Poisson-Boltzmann surface area (MM-PBSA) analysis was performed through GROMACS version 4.6.5 for the analysis of the conformational changes in the biomolecular protein-inhibitor complex (Aldeghi et al., 2016). The free energy calculations of the protein-inhibitor binding ΔG_*bind*_ is defined as the difference between the free energies of protein–ligand complex (ΔG_*cpx*_) and the unbound receptor/protein (ΔG_*protein*_) and ligand (ΔG_*lig*_) given as follows:

$$\begin{array}{l} \Delta G_{binding}=G_{complex}-\left(G_{protein}+G_{ligand}\right) \end{array}$$

The binding of the each inhibitor is to be evaluated through following parameters: the molecular mechanics potential energy comprising the bonded and non-bonded interactions such as angle, dihedral, electrostatic, and van der Waals; the free energy of solvation comprising of non-polar and polar energies (Kumari et al., 2014). The free binding energy for the particular ligand can be given with the following equation:

$$\begin{array}{l} \Delta G_{bind}=\Delta E+\;\Delta G_{solv}+\;\Delta G_{SA}\\ \Delta E=E_{complex}-E_{protein}-E_{ligand} \end{array}$$

where, *E*_*complex*_, *E*_*protein*_, and *E*_*ligand*_ are the minimized energies of the protein-inhibitor complex, protein, and inhibitor, respectively.

$$\begin{array}{l} \Delta G_{solv}=G_{solv\left(complex\right)}-G_{solv\left(protein\right)}-G_{solv\left(ligand\right)} \end{array}$$

where, *G*_*solv*(*complex*)_, *G*_*solv*(*protein*)_, and *G*_*solv*(*ligand*)_ are the salvation free energies of the complex, protein and inhibitor, respectively.

### Evaluation of ADME properties

The QikProp (Suite, 2014) package in Schrödinger suite was utilized for the evaluation and estimation of adsorption, distribution, metabolism, and elimination properties for two anti-ataxia compounds reported (Goyal S. et al., 2014). The modules predicts over 50 molecular properties such as molecular weight, central CNS, skin permeability (QPlogkp), free energy solvation, etc. indicating the safe assessments of these compounds in comparison with parity indicator for 90% drugs (Natarajan et al., 2015).

## Results

### Segmentation of data set and its validation

The hydromaxic based dataset comprising of the 44 derivatives (Table S1, Supplementary File) have been classified using the contingent culling method with the measure of 85% compounds in the training set. Thus, seven compounds viz., 8, 15, 26, 30, 32, 41, 43 (additional file 1) constitutes the test set (Golbraikh and Tropsha, 2002) and remaining 37 compounds represents training set. The test set was selected based on the criteria (Oprea et al., 1994) that all the representative compound points of the test set in the multidimensional descriptor space must be close to those of the training set (Dubey et al., 2008). Unicolumn statistics demonstrates the robustness of the selected training and test set (Table 1A). Data statistically demonstrated that the maxim of training set was greater than the maxim of test set and min of the training set was less than min of the test set, a condition which is prerequisite for a good QSAR model. The result showed that test set is interpolative i.e., derived within the max-min range of the training set. Further the mean and standard deviation of the training and test set points toward the relative difference of mean and point density distribution of the two sets.

**Table 1A:** **Unicolumn statistics for the training and test set**.

	**Average**	**Maximum**	**Minimum**	**Standard deviation**	**Sum**
Training	6.9721	8.0000	5.0400	0.6669	271.9100
Test	6.7920	7.5200	6.2600	0.4850	33.9600

### 3D QSAR model

Partial least square (PLS) analysis was employed in order to analyze the co-relation between the structure-activity having a pragmatic and rationale approach. PLS analysis is one of the standard statistical methods used for generation of predictive 3D-QSAR model. Gasteiger-Marsili charges were selected for computing force-field. Out of a total of 7148 3D molecular descriptors, 1233 obtained after eliminating the static rank contributed to the model generation. Since static strings are statistically similar and inefficient, hence they do not contribute to the model building. Only electrostatic and static descriptors contributed to the model generation (Potshangbam et al., 2011). Partial least square (PLS) regression analysis along with stepwise forward variable selection method was applied to build the 3D-QSAR model. Model obtained has been represented below with the equation:

(1) $$\begin{array}{rcl} pIC50 & = & 2.83451\left(E\_1657\right)+2.68845\;\left(S\_1911\right)\\ -\;3.97667\;\left(S\_1696\right)\;+\;2.80171 \end{array}$$

Here, three descriptors namely *E*_1657, *S*_1696, and *S*_1911 were selected, with E and S representing the electrostatic and steric field interactions respectively. As given in Equation (1) above, each of the electrostatic and steric molecular properties are associated with their respective statistical adjuvant and the regression coefficient has been shown as last statistical parameter. Internal and external validation of the developed model was performed through the leave-one-out (LOO) method. Validity of the QSAR model was ascertained through the following statistical factors:

$$\begin{array}{l} \text{Co-relation coefficient }r^{2}=0.6774,\text{ Pred}\_r^{2}=0.7570,\\ \text{Cross-validated correlation coefficient }q^{2}=0.6157,\text{ Pred}\_r^{2}\_\text{se}=0.2589,\\ \text{Low standard error value }r^{2}\_\text{se}=0.3839,\;q^{2}\_se=0.3190,\\ F\text{-}test=77.7093. \end{array}$$

A high *F*-test value of 77.7093 confirmed that model is 99% statistically significant having one in 10000 probability of failure while other relevant statistical parameters such as *Z*-score for *r*^2^, *q*^2^, and *pred*_*r*^2^ have been specified of its importance in QSAR-model in Table 1B. *Z*-score is the relative measure of the respective score deviation from the mean (μ) along with the robustness of model which can be ascertain with *Z*_score_*r*^2^, *Z*_score_*q*^2^, and *Z*_score_*pred*_*r*^2^-value of 7.15, 3.21, and 2.944, respectively.

**Table 1B:** **Statistical parameters for the 3D-QSAR model**.

**Dep Variable**	***Z*-score *r*^2^**	***Z*-score *q*^2^**	**Best rand *r*^2^**	**Best rand *q*^2^**	**Alpha rand *r*^2^**	**Alpha rand *q*^2^**	***Z*-score *pred r*^2^**	**Best rand *pred r*^2^**	**Alpha rand *pred r*^2^**
pIC_50_	7.15928	3.21294	0.27393	0.01881	0.00000	0.00100	2.94453	0.27293	0.01000

We also scaled the contribution of the molecular properties i.e., 3D descriptors as percentage contribution in the developed model. The grid points *E*_1657 and *S*_1696 have a positive contribution (28.538 and 37.189%) whereas the third descriptor *S*_1911 has a negative contribution (−15.748%) toward the inhibitory potential (Figure 3). Generally, steric descriptors are regarded as the bulky descriptors which relates to both size, shape, and fragments of the compounds. Thus, a steric descriptor having positive contribution represents the significance of a bulky group at that grid location. Steric descriptor S_1696 having its closeness with the bulky hydroxamic acid moiety signifies its presence at the active site as it enhances the anti-HDAC activity. The electrostatic descriptor on the other hand highlights the importance of electropositive and electronegative groups at a grid location. Electrostatic descriptors having positive contribution represents the importance of electropositive group whereas the one having negative contribution represents electronegative group (Alabed et al., 2016). E_1657 and S_1696 contribute positively, and are located near to the hydroxamic moiety. Thus, the presence of the electropositive group at R1 hydromaxic site is required as compared to electronegative group. The descriptor S_1911 has negative contribution thus the presence of bulky group decreases the activity (Figure 4). R1 aromatic ring must have non-bulky groups attached in order to enhance the activity, for which compounds 14 and 28 have 2-cyclopentyl and 2-hydroxy amino methyl attached at the 2nd position. Compounds 12 and 39 with non-bulky group 3-pyridazin-4-yl and 6-cyclopropyl pyridazin-4-yl at 3rd and 6th position, respectively, are few other examples.

### Pharmacophore hypothesis generation

Pharmacophore hypothesis applies the procedure of torsion sampling, through which the conformers minimized during the sampling are eliminated, having insignificant statistical parameters such as potential energy surface (Patel et al., 2002). Least Square Procedure (LSP) was applied for scoring the pharmacophore where the ligands were classified as per the alignment between the actives and pharmacophore features. Pharmacophore model was generated considering the inhibitors within the pIC_50_ activity threshold range of 6.5–7.5. Phase module of Schrödinger was used for the development of pharmacophore hypothesis. The module provides a variety of options in order to explore an array of models that are in line with the selected dataset. The models are generated through conformational sampling and scoring techniques. The scoring function comprises of a site score, vector score and volume score obtained with collective features for each aligned pharmacophore. Thus, the collective features of these separate lists yield vector score for each non-reference pharmacophore that have been aligned to the reference pharmacophore (Sun, 2008). Each compound is represented by separate site points or features which facilitates non-covalent binding between the compounds and its target receptor. Phase comprises of six build in pharmacophoric features—[1] Hydrogen bond acceptor (A), [2] Hydrogen bond donor (D), [3] Hydrophobic group (H), [4] Negatively ionizable group (N), [5] Positively ionizable group (P), [6] Aromatic ring (R). Apart, from these six pharmacophoric features the module provides three custom features in order to accommodate the characteristics that does not fit in the default build in categories (Jones et al., 1995). A total of 28 variants were generated keeping five as maximum and four as minimum number of sites. The pharmacophore hypothesis generated along with their survival scores and selectivity are reported in Table 2A. The scoring of each of these hypotheses was carried out using the ligand classification as per the alignment between the reference and non-reference pharmacophore. A score is assigned to pharmacophore from confined box regarded as reference and further all non-reference pharmacophore are aligned to the reference pharmacophore. The alignments are measured by two criteria, Root-mean-square deviation (RMSD) in site point position and Average cosine of angles formed by corresponding vectors such as acceptor, donor, and ring aromatic. The corresponding vector score and site score can be derived through following equation:

$$\begin{array}{l} Site\_Vector\_Score_{i}=w_{Site}SiteScore_{i}+w_{vector}VectorScore_{i} \end{array}$$

Where

$$\begin{array}{l} Site\_Score_{i}=1-\frac{RMSD}{cut-off_{RMSD}}\\ Vector\_Score_{i}=1/{\text{n}}_{\text{v}}+\overset{n_{v}}{\underset{j=1}{\sum }}cos\theta_{ij} \end{array}$$

**Table 2A:** **Different pharmacophore hypothesis generated for virtual screening**.

**Row**	**ID**	**Survival**	**Survival-inactive**	**Selective**	**Matches**
1	DDHHR.2	3.789	1.220	1.564	25
2	DDHHR.37	3.584	1.162	1.595	25
3	ADDRR.5	3.712	1.161	1.598	25
4	ADDRR.8	3.789	1.220	1.564	25
5	**ADDRR.20**	**3.846**	**1.230**	**1.597**	**25**
6	ADDRR.53	3.832	1.242	1.594	25
7	ADDRR.55	3.695	1.175	1.593	25
8	ADDRR.67	3.832	1.242	1.594	25
9	ADDRR.70	3.695	1.175	1.593	25
10	DHHRR.15	3.846	1.230	1.597	25
11	DHHRR.19	3.712	1.161	1.598	25
12	ADHRR.42	3.675	1.195	1.594	25
13	ADHRR.51	3.813	1.263	1.596	25

**Table 2B:** **Predicted activity of the top scoring compound using the selected pharmacophore hypothesis**.

**Compound ID**	**XP-score**	**Align score**	**Vector score**	**Volume score**	**Fitness**	**Predicted activity (through 3D-QSAR model)**	**Binding interaction**
Zinc 00608101	−**10.09761**	**1.949571**	**0.829256**	**0.347826**	**1.179391**	**7.53**	**(His802-Asp838) catalytic dyad**
Zinc 19702930	−10.02283	1.251246	0.738099	0.415301	1.084544	7.22	Pro676, Arg681
Zinc 20464210	−10.07625	1.203255	0.673085	0.42716	1.115548	6.94	Asp934, His976
Zinc 00329110	−**9.182012**	**1.037448**	**0.612613**	**0.420593**	**0.938919**	**6.84**	**(His803-Asp840) catalytic dyad**
Zinc 00897385	−9.182680	1.401166	0.816804	0.335443	1.102823	6.82	No interaction
Zinc 20465875	−9.769430	1.591722	0.669213	0.41623	0.718525	6.81	His131-Asp166
Zinc 00518218	−9.769608	1.081293	0.507579	0.431319	1.298446	6.08	No interaction

*The bold values represent the selected entity*.

The parameters *w*_*site*_, *w*_*vector*_, and *cut-off*_*RMSD*_ are user defined with default values of 1.0, 1.0, and 1.2, respectively. n_v_ is the number of vector features in the hypothesis and θ_*ij*_ is the angle between the *j*th vector feature in the non-reference pharmacophore and the corresponding vector feature in the reference pharmacophore.

The reference hypothesis is the one with the highest score which represents the confined box having all the pharmacophores. Consequently, the ligand which contributes to the reference pharmacophore is referred to as reference ligand; once the scoring hypothesis is selected the low scoring ones are eliminated, such that only hypotheses in the top 10% are retained (Debnath, 2002). They are refined through volume scoring, selectivity scoring and number of actives matched based on Vander Waals model of structure through the alignment of reference and non-reference ligand.

A common pharmacophore hypotheses is defined as pharmacophore derived from all conformations of the active ligand having resemblance to the bound ligand features and spatial arrangement. Thus, the hypothesis for each box (representing a common pharmacophore) was chosen. After selecting the hypothesis for the each box, final scoring was computed, and the resultant score known as survival score of hypothesis which determines its validity and potential was used for given set of molecules as can be seen below:

$$\begin{array}{lll} S & = & W_{site}S_{site}+W_{vec}S_{vec}+W_{vol}S_{vol}+W_{sel}S_{sel}+W_{rev}^{m}\\ -W_{E}\Delta E\;+W_{act}A \end{array}$$

Here, *W* is weight and *S* is score.

On the basis of survival scores for actives and inactive we identified a pharmacophoric hypothesis demonstrating maxim five and minim four sites aligned with that of active compounds of the congeneric dataset. ADDRR.20, a pharmacophore hypothesis implies the presence of one hydrogen acceptor, two hydrogen donor and two ring aromatics. ADDRR.20 had an optimum alignment with active set of compounds and demonstrates high selectivity along with a survival score of 3.846, survival inactive score of 1.230, active score of 0.9940, vector score of 1.0000, and volume score of 0.8544.

The selected pharmacophore hypothesis (ADDRR.20) indicates the presence of two hydrogen donors (D), two ring aromatics (R), and one hydrogen acceptor (A). All these features signify the mode of binding and in screening of compounds through various chemical libraries. Figure 5A represents the alignment of compounds in the selected pharmacophore along with pharmacophoric features such as, the hydrogen donors (blue), spherical with arrows indicating toward the H-bond. The aromatic rings are presented through orange rings with the centroid of the atoms and the single hydrogen acceptor has been demonstrated in light red. Figure 5B demonstrates the alignment of active compounds for the chosen hypothesis ADDRR.20 and the inter-site distance among the pharmacophoric features, respectively.

### Model validation of selected pharmacophore and 3D-QSAR model

**Figure 1:**
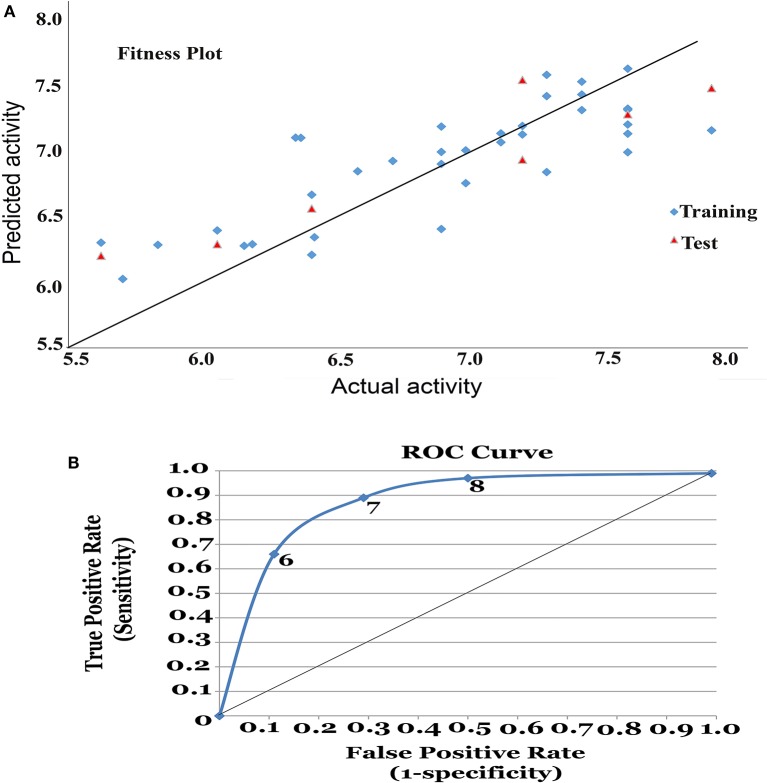
**(A)** Fitness plot for the training and test set. **(B)** Receiver Operating Characteristic (ROC) curve for 3D-QSAR model.

The model generated was validated before employing it for screening the combinatorial drug libraries and predicting their activities. Thus, both models were validated based on different statistical parameters. The selected pharmacophore model was validated to check its ability to identify known actives from the dataset of the known inactive i.e., GH and EF score (Table 3A). In order to do this, the selected pharmacophore model was imputed to screen the dataset of known active (A, 4930) and inactive molecules (77324). The selected model retrieved 5689 hits including 4437 known actives (78.18%, yield). The enrichment factor (EF) of this screening protocol was calculated to be 13.012, which indicated that selected model has 13 times more stability to identify active molecules than inactive. The goodness of hit score (GH) of this screening protocol was calculated to be 0.796 which indicates the significance of selected pharmacophore to identify active molecules. Further, an external dataset comprising of the known HDAC inhibitors (Librizzi et al., 2016; Patel et al., 2016; Wen et al., 2016; Zhu et al., 2016) that were not included in QSAR model generation and were used as external test set were selected for validating the predictive power of the developed QSAR model. The robustness of the developed QSAR model was analyzed by performing “Y-randomization” test, the low value of $R_{scramble}^{2}$ (0.629) as compared to $R_{train}^{2}$ of selected model, described the real regression correlation of the selected model. In addition, the calculated values of *k* (1.003), *k*′ (0.951), $R_{\text{o}}^{2}$ (0.984) and $R_{\text{o}}^{’2}$ (0.993) were also within the recommended range that strength the external predictive ability of the selected 3D-QSAR model. The developed QSAR model was further validated through receiver-operating characteristic (ROC) curve (Table 3B). The curve analyses the ability of a particular model to correctly classify a list of compounds as actives or inactive and is indicated by the area under the curve. The area under the curve (AUC; Figure 1B) was calculated as 0.8157. Thus, we can conclude that our model is not randomly classified considering area under the ROC curve is statistically significant from those obtained by random classifier (area = 0.5).

**Table 3A:** **Statistical parameters for the calculation of Goodness of hit score (GH) and Enrichment Factor (EF)**.

**S.No**	**Parameters**	**Values**
1	Total molecules in database (D)	82254
2	Total Number of Actives (A)	4930
3	Total hits (Ht)	5689
4	Active hits (Ha)	4437
5	% Yield of Actives [(Ha/Ht) × 100]	78.01
6	% Ratio of Actives [(Ha/A) × 100]	90.05
7	Enrichment Factor (E) [(Ha × D) / (Ht × A)]	13.012
8	False negatives [A − Ha]	493
9	False positives [Ht − Ha]	1252
10	Goodness of hit score (GH)^a^	0.796

a *[(Ha/4HtA) (3A + Ht) × (1 − ((Ht − Ha) / (D − A)))]; GH score of >0.7 indicates a statistically good model*.

**Table 3B:** **ROC curve cut-off values along with their respective true positive rate and false positive rate**.

**S.No**	**Cut-off**	**True positive rate sensitivity (Se)**	**Specificity (Sp)**	**False positive rate (1-specificity)**
1	6	0.66	0.89	0.11
2	7	0.89	0.71	0.29
3	8	0.97	0.50	0.50

### Pharmacophore based virtual screening using docking and molecular dynamics for predicting their inhibitory activity against HDAC4

The generated pharmacophore hypothesis ADDRR.20 comprising of five pharmacophoric sites was utilized for screening a repository of ~82,000 compounds, resulted in a total of 4930 hits, with four out of five must match criteria. These screened compounds were further analyzed for their binding pattern and mode of interaction with HDAC4 (PDB ID: 4CBY) using HTVS and XP docking protocol of Glide. The compounds demonstrating the binding affinity of more than –8.0 kcal/mol (in magnitude) through HTVS were docked again with HDAC4 (PDB ID: 4CBY) in order to re-affirm the binding pattern and its affinity between the docked position of the selected molecules and naïve X-ray crystal structure using the XP protocol (Halgren et al., 2004). The XP-score signifies the binding affinity of the respective lead compounds with HDAC4 catalytic dyad consisting of two histidine aspartic dyads. Thus, on the basis of binding affinity and mode of interactions, two compounds, the first compound ***[1-((2S)-2-[(2-chlorothiophen-3-yl)methoxy]-2-(2,4-dichlorophenyl)ethyl)-2,3-dihydro-1H-imidazole]*** (Figure 6A) (**SEI**, ZINC 00608101) possessed a docking score of -10.097 kcal/mol and the second compound ***[3-((2S)-2-amino-3-[(furan-2-ylmethyl)amino]-3-oxopropan-3-ium-1-yl]indol-1-uide*** (Figure 6B) (**ACI**, ZINC 00329110) demonstrated a docking score of –9.182 kcal/mol were selected for further study. The generated QSAR model was utilized for predicting the activities of top scoring compounds screened through common pharmacophore hypothesis. High predicted activities of SEI (7.53) and ACI (6.84) further re-assert the inhibitory potential of these compounds against HDAC4. The predicted activities of the screened compounds from database where scored through the 3D-QSAR model and re-ranked accordingly as shown in Table S2 (Supplementary Data). The docking scores and predicted activities of the top scoring compounds have been further summarized in Table 2B. We observed the interaction pattern for the other screened compounds such as ZINC 518218 and ZINC 00897385 as shown in Figure 7. The interactions of the two top scoring compounds were analyzed with the catalytic dyad (His802-Asp838 and His803-Asp840) through molecular dynamics simulations for a time period of 30 ns (Figure 7B). The first compound SEI (Figure 7A-a) having a bulky ring structure showed hydrophobic interaction with the catalytic dyad along with other residues: Leu943, Asp934, His802, Pro800, Arg681. The structure demonstrated stability for a time period between 5 and 15 ns during MD simulations. The complex again stabilizes around 20 ns in accordance with the apo-form of the HDAC4 protein up to 30 ns. The second compound ACI (Figure 7A-b) demonstrated hydrophilic interactions with catalytic dyad apart from Asp936, His976, Pro676, Glu677, Phe812, Asp759, His803, and His842. The inhibitor was not in the common plane of the His131-Asp166 pair and aligned to a horizontal position being 3.5 Å from the imidazole ring resulting in the open conformation. However, around 15 ns the inhibitor exhibits a closed conformation through interactions with the loop regions between the residues Pro676, His842, Phe812 as well as Leu943 and Asp936. The complex HDAC-SEI demonstrated having stable orientation and binding pattern with respect to HDAC4-ACI complex. The RMSD graph (Figure 8A) demonstrates the molecular dynamics simulations course performed with respect to RMSD vs. time for HDAC4 crystal structure (apo-form) shown in purple, the HDAC4-SEI complex shown in blue, and the HDAC4-ACI complex shown in orange. The large scale fluctuations in the RMSD graph (Guo et al., 2010) represents the absence of steric influence due to non-bulky aromatic group resulting in loose interaction of ACI with the catalytic dyad throughout up to 30 ns. Analysis of the post dynamics interactions of hydroxamic acid derivatives with HDAC4 revealed that the binding orientation of both the docked complexes was different with each other i.e., the complex HDAC4-SEI demonstrated similar binding orientation as the experimental binding mode (PDB ID: 4CBY) while HDAC4-ACI complex exhibited deviation in comparison to the experimental binding mode (Goodford, 1985). We also analyzed the potential energy map for the protein-inhibitor complex along with the apo-form of the HDAC4 protein for a time period of 30 ns (Figure 8B). It was observed that SEI inhibitor complex was stable in comparison to the ACI inhibitor complex as it was in accordance with the apo-form of HDAC4 protein with respect to conformational changes as well as in terms of energy.

### Free energy calculation for SEI and ACI

Molecular dynamics simulations were used to calculate binding free energy using MM/PBSA method. Snapshots were extracted at every 10 ns of stable intervals from 11 to 12 ns MD trajectory. The binding free energy and its corresponding components obtained from the MM/PBSA calculation of the protein-inhibitor complexes are listed in Table 4. The results indicated that SEI possessed negative binding free energy value of −1683.55 kcal/mol followed by ACI with value of −1591.90 kcal/mol. Moreover, van der Waals and electrostatic interactions and non-polar solvation energy negatively contribute to the total interaction energy while only polar solvation energy positively contributes to total free binding energy (Wang et al., 2016). In terms of negative contribution, van der Waals interaction gives much larger contribution than electrostatic interactions for all the cases. The non-polar free energy contributes relatively less as compared to the total binding energy (Verma et al., 2016). This indicates that non-polar solvation energy, van der Waals, and electrostatic interaction together contribute to the SEI-HDAC4 complex stability. The solvation energy comprising of the polar and non-polar free energies were found to increase while the electrostatic energy contributes to the enhanced binding affinity (Genheden and Ryde, 2015) of SEI compared to the ACI. In class IIa HDAC4 protein tyrosine is mutated to histidine Y967H with longer side chain resulting in the closeness of the interacting residue and increasing electrostatic interactions thereby enhancing the electrostatic energy. Further, calculation of the binding energies elucidated that the protein-ligand interactions for SEI was better than ACI. SEI demonstrated interactions with His803, Pro800, Glu677, Asp934, and Phe812 residues of the catalytic dyad whereas ACI was observed to interact with Asp936, Pro942, Phe871 and Arg861. This further illustrates that SEI-HDAC4 complex showed better free binding energies and higher interactions than ACI complex.

**Table 4:** **The binding free energies for SEI and ACI complex**.

**S.no**	**Anti-ataxia leads**	**MM P.E. (kcal/mol) (Electrostatic and van der Waal's)**	**Solvation Energy (kcal/mol)**		**Free binding energy (kcal/mol)**
			**Polar**	**Non-polar**	**Δ*G*_binding_**
1	SEI	−2229.795	560.762	−14.522	−1683.555
2	ACI	−2173.816	595.772	−13.857	−1591.901

### ADME evaluation for the compounds selected i.e., SEI and ACI

A total of 50 properties were analyzed using the QikProp module of Schrodinger software. Over the time, it has been referred that staunch prognosis of ADME properties acts as precursor screening tool for screening drugs and designing de-novo combinatorial libraries. In current study we chose a total of 50 descriptors having physio-chemical and pharmacokinetic molecular properties. These properties such as #star, CNS, blood-brain barrier coefficient (QplogBB), solvent accessible surface area (SASA), FISA, FOSA, skin permeability (Qplogkp), solubility (logP), molecular weight, free energy of solvent in hexadecane and water as QPlogPC16 and QPlogPoct respectively, ionization potential and others have been reported along with their respective values for SEI and ACI in Table S3 (Supplementary Data) and were found to be well within the reference range. Both the anti-ataxia compounds demonstrated drug like features with SEI being less toxic in comparison to ACI.

## Discussion

In current study, we applied 3D-QSAR model generation along with common pharmacophore hypothesis approach toward 61 diarylcyclo-propane hydroxamic dataset having anti-HDAC4 activity. Despite of the fact that computational approaches toward the role of HDACi against ataxia is relatively young, sufficient data are present to indicate the possibility to discover and design novel HDAC inhibitors using pharmacophore based virtual screening approaches. An increasing large number of HDACi are being reported, chemo-informatical analyses of these reported HDACi allows researchers to analyze the chemical space occupied by HDAC's and to create filters that can be included in virtual screening experiments together with pharmacophores (Tang et al., 2009; Ganai et al., 2015; Zhou et al., 2015).

### Analysis of 3D-QSAR model

3D-QSAR model aims to statistically co-relate the alternatives in chemical structures and their respective biological activity through considering the molecular properties i.e., steric, electrostatic and hydrophobic shown on the spatial grid location. It thereby correlates the non-bonded interactions fields with that of the biological activity (Akamatsu, 2002). The equation of the developed QSAR model had three physicochemical descriptors, one electrostatic, and two steric descriptors. The first descriptor, *E*_1657, belonged to electrostatic class. It signifies the presence of electropositive groups as compared to electronegative groups. The contribution plot showed a positive contribution of 28.538% for this descriptor, which indicated that compounds should have electropositive substitution at R1 grid location so as to have an improved inhibitory activity. Next descriptor, *S*_1911 is a type of steric descriptor which in this model has a positive contribution of 37.189%. This implied that presence of a bulky group at R2 grid location increases the inhibitory activity of the lead molecule. Hence, a compound without steric hindrance at its R2 grid location would not have a better inhibitory effect in comparison to a group having a bulky group causing steric hindrance. The third descriptor, *S*_1911, is a type of steric descriptor with a negative contribution of 15.748% signifying the presence of steric hindrance with bulky groups as limiting factor toward the inhibitory activity. Descriptors signify the molecular properties essential for the increased inhibitory activity of the compounds (Almerico et al., 2010). Thus, the descriptors with positive contribution enhance the inhibitory effect while the descriptor with negative contribution decreases the inhibitory effect. The statistical values obtained in the 3D-QSAR model showed 67.74% variance in the observed activity values of the compounds in training set, and low standard error of *r*^2^_se = 0.2589 demonstrated the accuracy of the model. The *F*-test value of 77.7093 represented a good overall statistical significance level, which means that the probability of failure of model is very less. Cross-validated correlation coefficient (*q*^2^) judged by leave-one-out method had a value of 0.6157 and indicated a good internal prediction power of the model. Furthermore, a good external predictive potential of the model was estimated by high *pred*_*r*^2^, in this case 0.7570. Low standard error values indicated absolute quality of the model. Thus, it can be deduced that the model is reliable and predictive.

**Figure 2:**
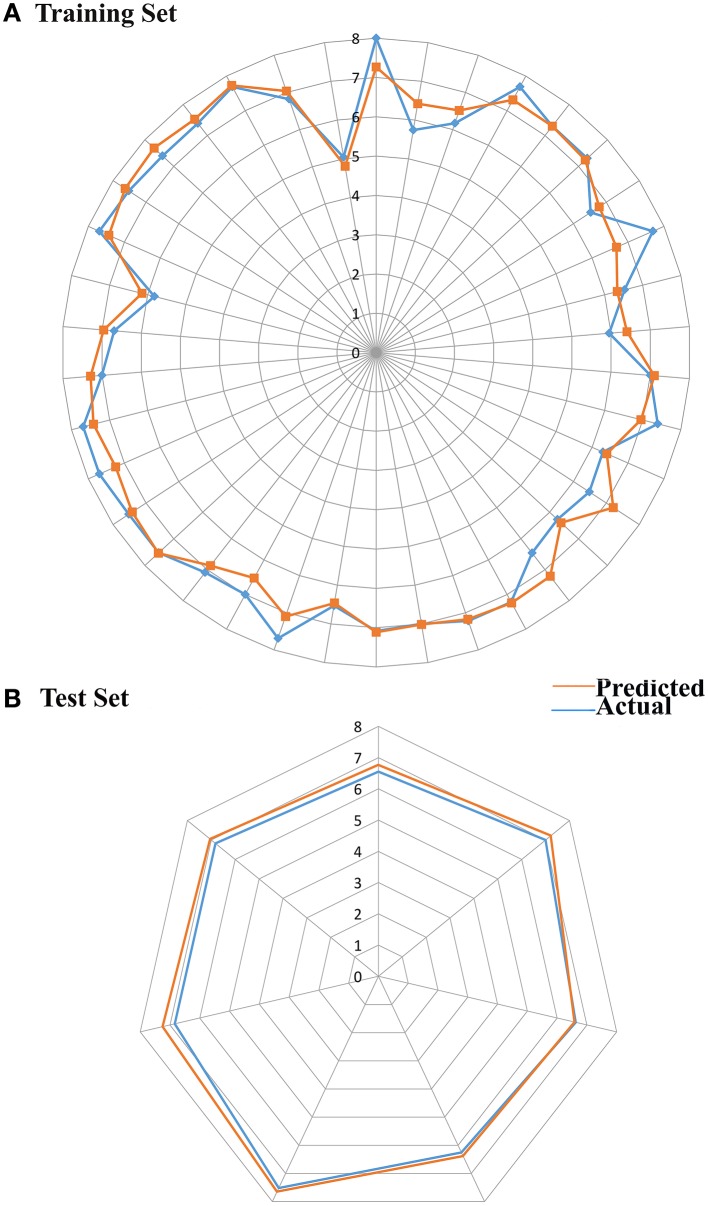
**Radar plots demonstrating actual and predicted value for (A)** Training **(B)** Test set.

From the above observations, we can conclude that the substitution of electropositive groups at R1 grid location enhances the inhibitory activity, whereas the presence of non-bulky group at R2 would lead to lower inhibitory activity. Further, graphical representation between the actual and predicted activities of the 44 compounds has been demonstrated through the data fitness plot (Figure 1A), whereas the graphical representation for the test and training set has been shown through the radar plots. Data fitness plot represents the co-relation between the actual and the predicted values as greater the deviation from the regression line, more is the difference between the actual and the predicted values (Cramer et al., 1988). On the other hand, radar plot (Figure 2) depicts the extent of overlapping between the test and the training set through the overlap between the blue (actual activity) and red (predicted activity). Radar plot statistically validates the robust nature of the developed QSAR-model i.e., a radar plot showing high value for *r*^2^ and *pred*_*r*^2^ for training and test set respectively demonstrates good overlap between actual and predicted activities. Another reliable metric to evaluate the performance of the 3D-QSAR model is the AUC of the ROC curve (Figure 1B). The present model achieved a good value of 0.815719 AUC thereby concluding that the model is not randomly classified (Table 3B).

**Figure 3:**
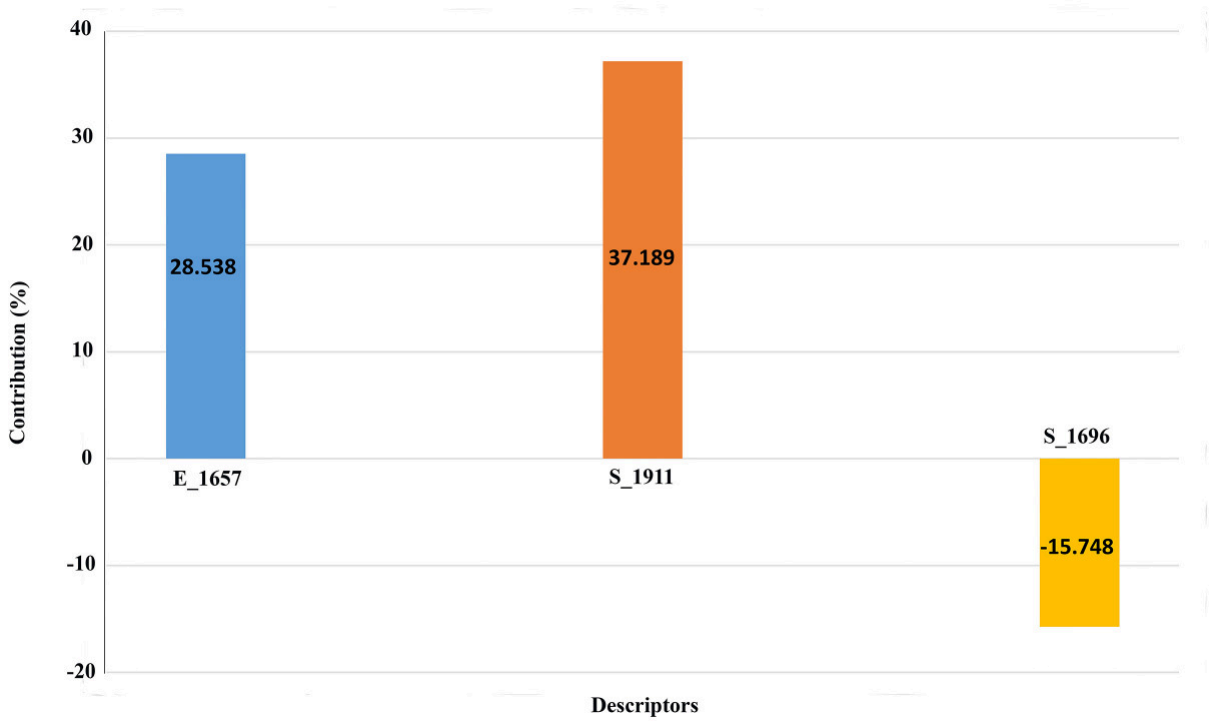
**Contribution plot for the selected molecular properties in 3D-QSAR model**.

**Figure 4:**
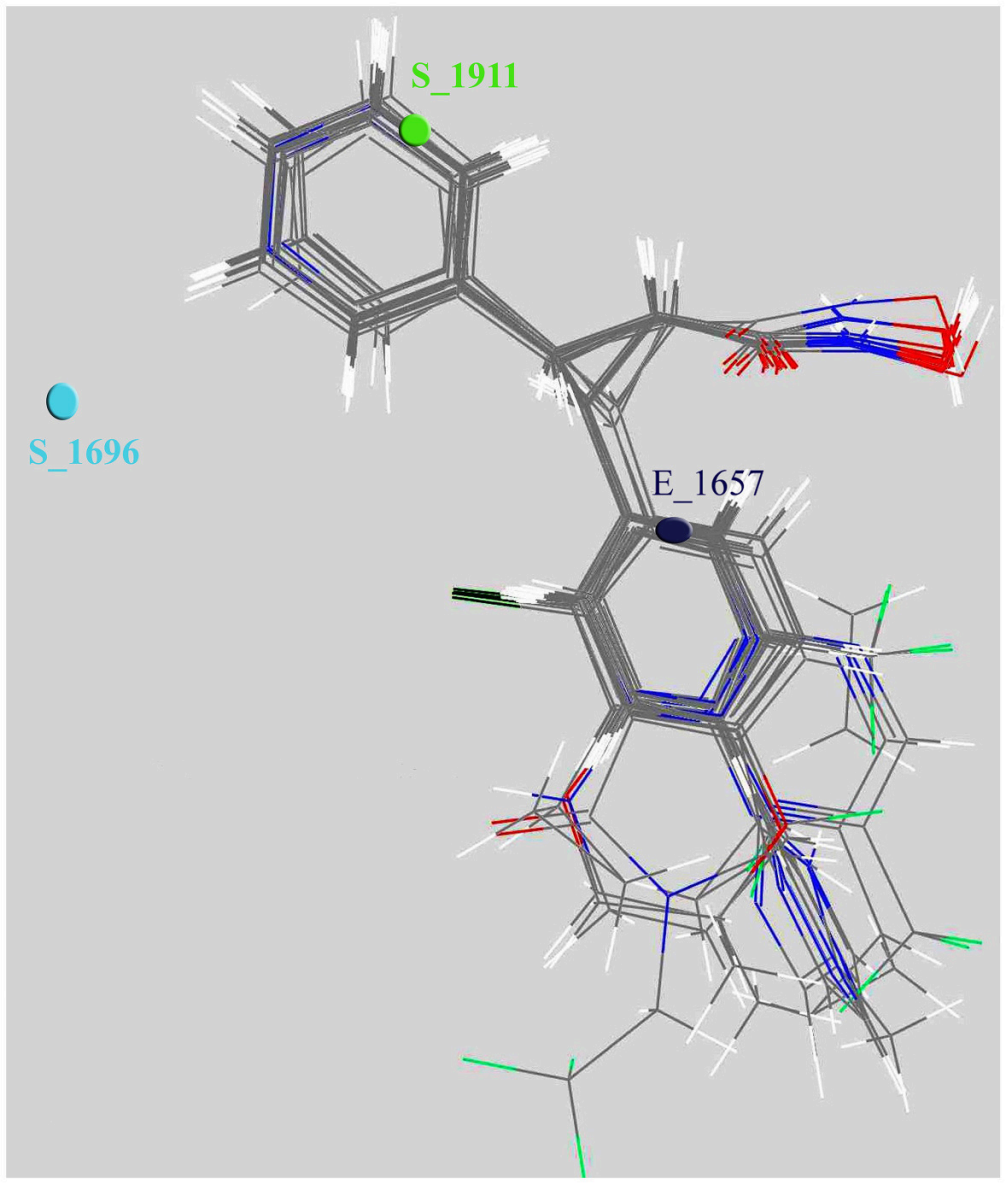
**Depiction of aligned congeneric set of molecules and 3D descriptors marked in the cubic grid**.

**Figure 5:**
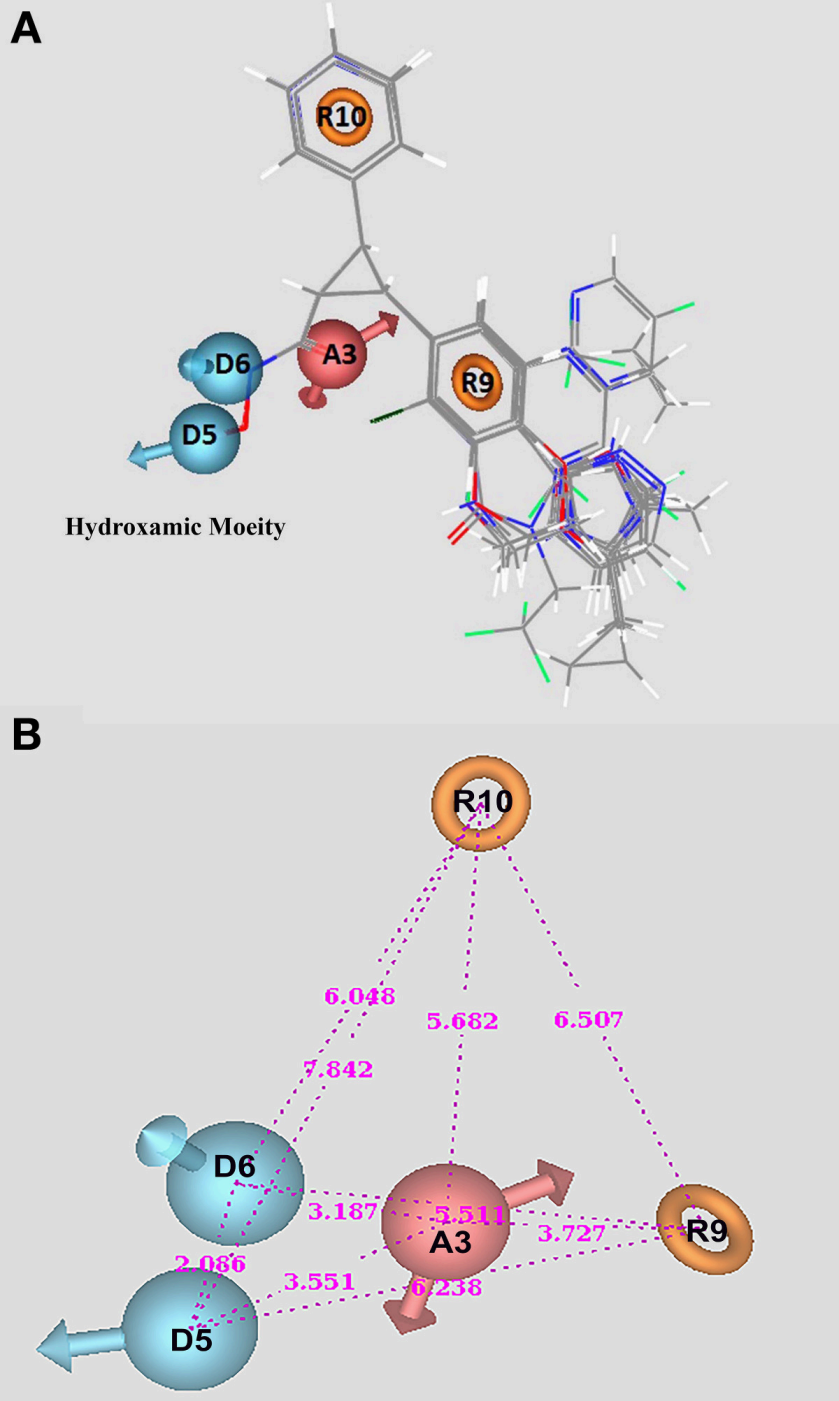
**Hydroxamic based HDAC inhibitors marked with pharmacophore features of ADDRR.20**. **(A)** Alignment of molecules along with pharmacophore features. **(B)** Intersite distance between pharmacophore sites.

### Pharmacophore and structural analysis of selected leads with HDAC4

**Figure 6:**
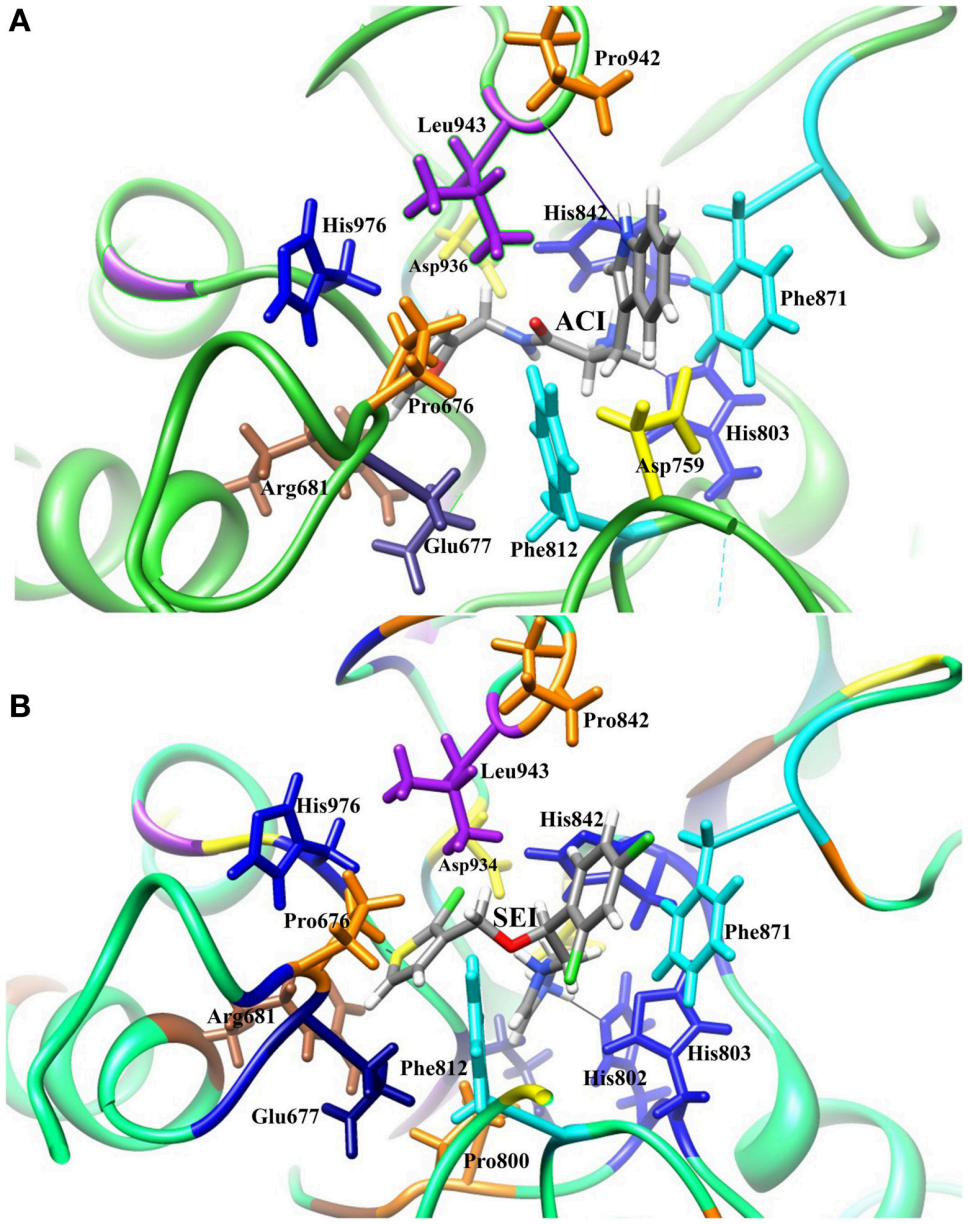
**Interactions between HDAC4 protein and selected compounds (A)** ACI **(B)** SEI.

The pharmacophore model ADDRR.20 comprising of five pharmacophoric features i.e., a hydrogen bond acceptor (HBA), two hydrogen bond donor (HBD), and two ring aromatic (RA) was utilized for identifying potential new actives through ligand alignments (Hu and Lill, 2014) for subsequent database screening with minimum four out of five features (Caporuscio and Tafi, 2011). The identified leads (SEI and ACI) having the fitness score of 1.0 or above based on pharmacophore screening were investigated for further structure activity insights. The binding mode for compound SEI with active site residues showed that mapped on hydrogen bond donor (HBD) feature of ADDRR.20 it formed interactions with Arg681, Pro676 whereas hydrogen bond acceptor (HBA) feature of ADDRR.20 forms interaction with His802, His 803, Phe812, and Pro800. Further, binding mode analysis for SEI demonstrated that HBD feature mapped on NH, HBA mapped on O along with imidazole and thiopene mapped on ring aromatic (RA) forming interactions with active site residues. The compound ACI formed hydrogen bond interactions with Asp759, Phe871, His803, Leu943, and Pro942 as shown in Figure 6. The binding mode and pharmacophore overlay of the ACI demonstrates that O mapped on the HBA, NH mapped on HBD along with 1H-indole and furan mapped on RA features forming interaction with amino acid residues. Thus, the study indicates that His 802, Asp934, His802, Leu943, Arg681, Phe812 were the key amino acids residues in the active site involved in hydrogen bond interactions of HDAC4 with selected molecules (SEI and ACI) which aligns with recent studies on HDACi binding pattern (Ragno et al., 2006). Our pharmacophore model ADDRR.20 was also in alignment with the previous developed models toward histone de-acetylases (HDAC's) with known hydroxamic acids and cyclic peptides having four pharmacophore features i.e., one hydrogen bond acceptor, one hydrophobic group, and two aromatic rings (Vyas et al., 2016) and also with known hydroxamic acids, benzamides, and biphenyl derivatives on HDAC8 (Vadivelan et al., 2008) having three pharmacophore features i.e., hydrogen bond acceptors, hydrogen bond donors, and hydrophobic aromatic ring. Thus, our developed pharmacophore model with known hydroxamide derivatives toward HDAC4 having five pharmacophore features i.e., one hydrogen acceptor (HBA), two hydrogen donor (HBD), and two aromatic rings (RA) correlates with the previous studies (Thangapandian et al., 2010a). The pharmacophore model ADDRR.20 comprises of features for inhibitor-protein interaction, crucial for its binding and biological activity which is evident through its binding mode and pharmacophore overlay of the selected molecules (SEI and ACI), validating the significance of its features and the model. Further, to validate the discriminatory ability of the ADDRR.20 model, we retrieved 82,000 decoy molecules from chemical databases. ADDRR.20 model was able to find 100% of active compounds in the hit list. We calculated EF for the generated models to estimate the contribution of the active molecules ranking. For the hypothesis ADDRR.20, EF-value was 13.012 indicating the superiority of the pharmacophore model (Thangapandian et al., 2010b) ranking over random distribution (Table 3A).

**Figure 7:**
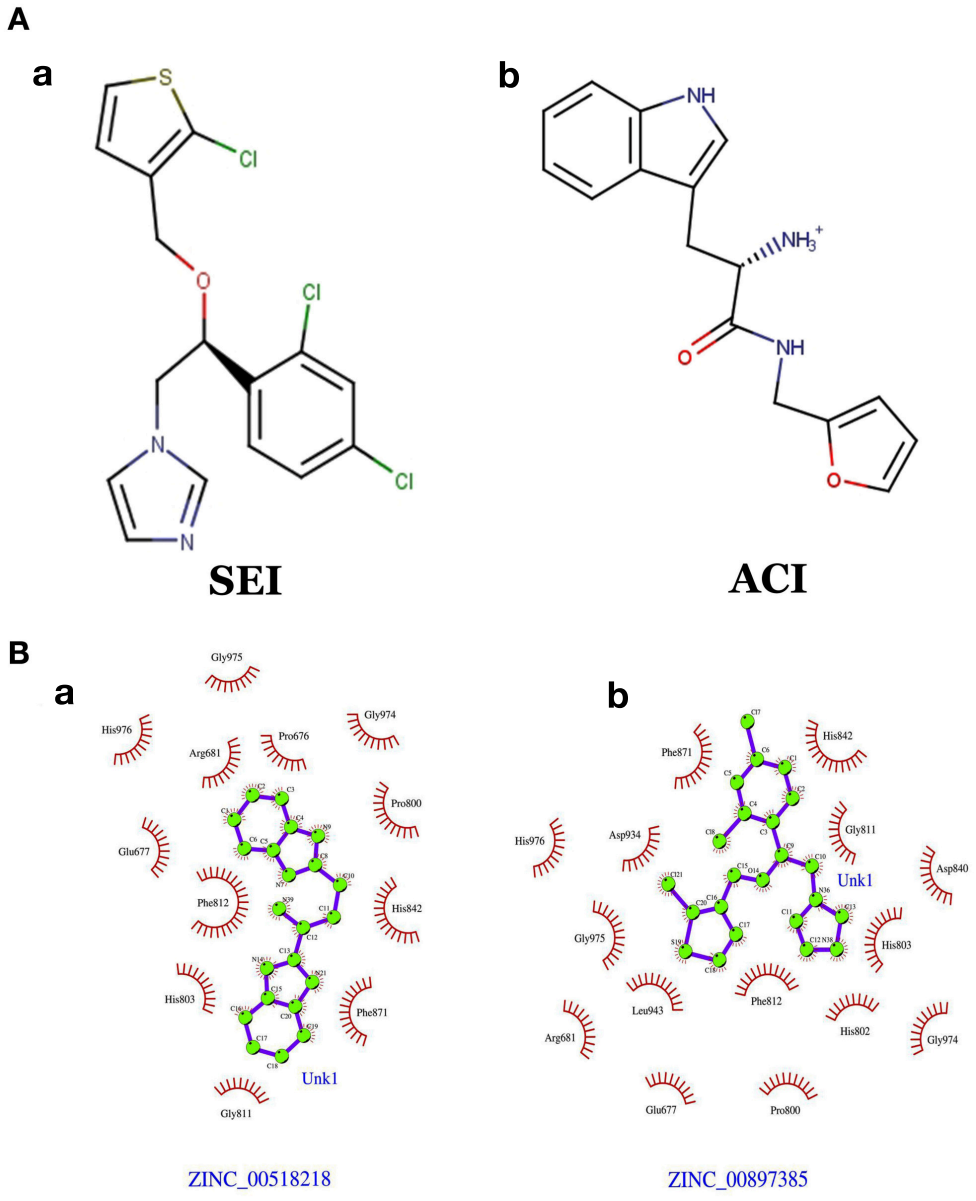
**(A)** Chemical structures of **(a)** SEI **(b)** ACI. **(B)** Interactions pattern between the HDAC4 protein for the screen molecules **(b)** ZINC 00897385 and **(a)** ZINC 005182189, respectively.

**Figure 8:**
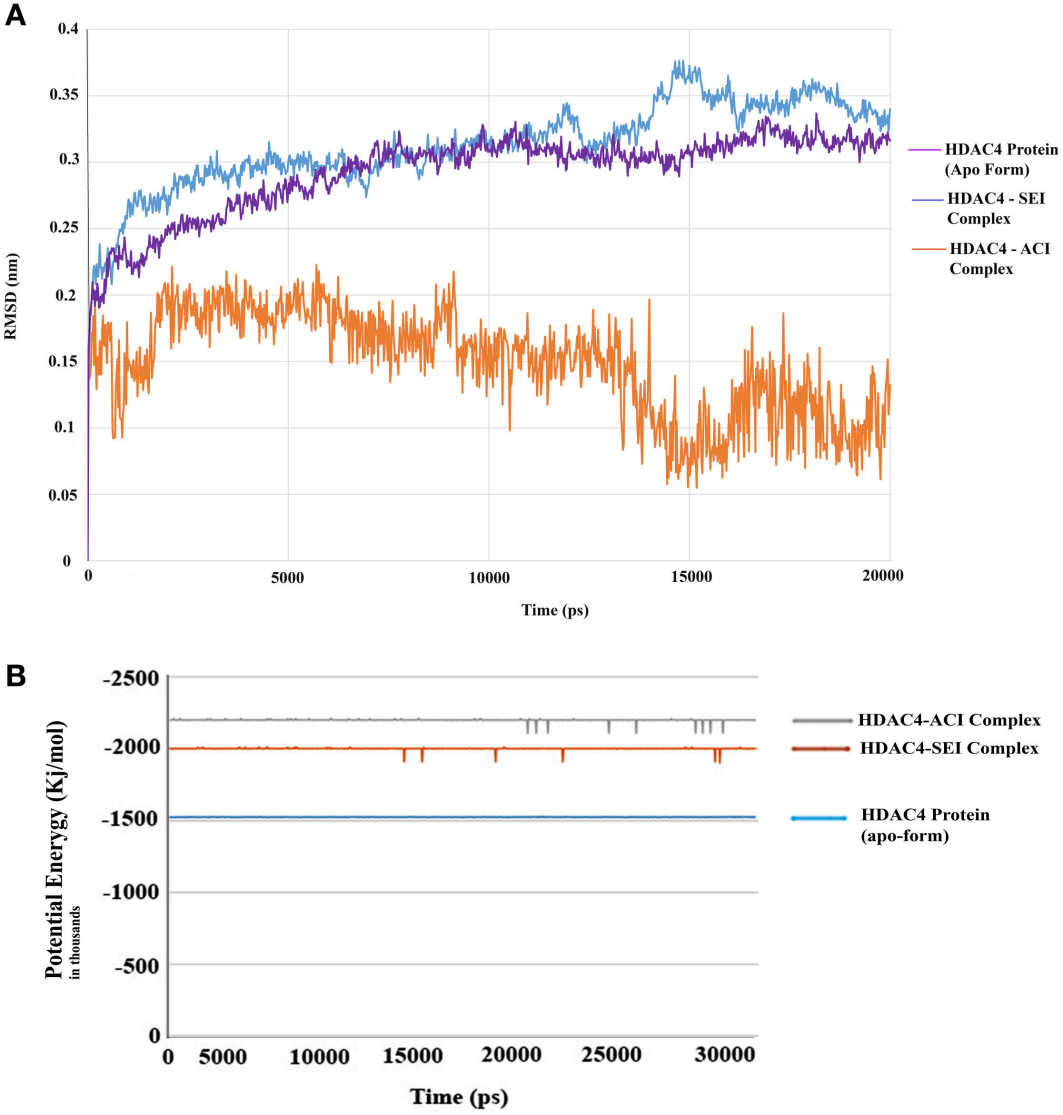
**(A)** RMSD graph for the selected compounds for SEI and ACI for a time period of 20 ns. **(B)** Potential energy graph for selected compounds SEI and ACI for a time period of 30 ns.

We also analyzed ADME properties of SEI and ACI through Qik prop, we have mainly focused on CNS, QPlogBB, QPlogkp, QPlogPo/w, and others (Table S3). If the molecules demonstrated the value as 0, −3.0 to 1.2, −1.7 to 1.6, −2 to 6.5, then it represents that the molecules have good central nervous system activity, blood-brain partition coefficient, skin permeability factor and predicted octanol/water partition indicating hydrophobic nature of the chemical compounds. Lipinski rule of five suggested that a chemical compounds could be an orally active drug in humans. The rule states that the most “drug-like” compounds should have the value of clogP ≤ 5, molecular weight ≤ 500, and number of hydrogen bond acceptor ≤ 10 and donors ≤ 5. The molecules SEI and ACI were found to be satisfactory in all the parameters, when evaluated on the grounds of Lipinski rule-of-five and Jorgensen rule-of-three violation.

## Conclusion

HDAC inhibitors comprising of hydroxamic moiety have been validated to be a promising novel targets for the treatment of solid tumors and hematological cancers but off-late their role has also been established toward polyglutamine disorders such as Huntington's. Thus, our study is guided with the ongoing effort toward the discovery and selection of the Hydroxamic based HDACi against ataxia. The purpose of this study was to utilize the generated five point pharmacophore model for identification and selection of the lead molecules from chemical databases as potential HDAC4 inhibitors. Thus, on the basis of 3D-QSAR, pharmacophore modeling, virtual screening, docking, and MD simulation study the molecules SEI and ACI were selected as novel leads for effective HDAC4 inhibition. Further, SEI can be examined as an anti-ataxia agent as it shows high binding affinity and stable dynamics, high free energy binding and has been found to be less toxic compared to ACI. One of the major limitation of the study is the experimental validation of the selected leads i.e., SEI and ACI, which we strongly believe can be achieved as the study provided valuable insights for further investigation through fluorescence spectroscopy/HDAC screening kit.

## Author contributions

The work has been primarily done by SS, at the later stages SG reviewed the manuscript and prepared the images. The work has been done in the supervision of PS and AG. All the agreement will be signed by AG.

### Conflict of interest statement

The authors declare that the research was conducted in the absence of any commercial or financial relationships that could be construed as a potential conflict of interest.

## Footnotes

^1^https://www.chemaxon.com/products/marvin/marvinsketch/

^2^Mds, V. VLife MDS software package, version 3.5, Pune, India 411007.
