# Supplementary material for: Mechanistic Insights into the Binding of Class IIa HDAC Inhibitors toward Spinocerebellar Ataxia Type-2: A 3D-QSAR and Pharmacophore Modeling Approach
Source: Front Neurosci. 2017 Jan 10;10:606. doi: 10.3389/fnins.2016.00606 (PMC5223442; doi:10.3389/fnins.2016.00606)
Supplement: Supplementary file 1 [file DataSheet1.DOCX]

| Template:  (1S,2R,3S)-N-hydroxy-2-methyl-3-phenylcyclopropane-1-carboxamide. | | | 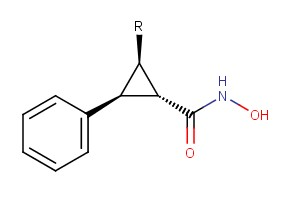 | | |
| --- | --- | --- | --- | --- | --- |
| S.No  **Supporting information**  **Table S1**: Fragments of di-aryl cyclo-propane hydroxamic acid derivatives along with the actual and predicted activity against HDAC4. | Name | Structure | | Actual activity  (pIC50) (µM) | Predicted activity |
| 1. | S1 | 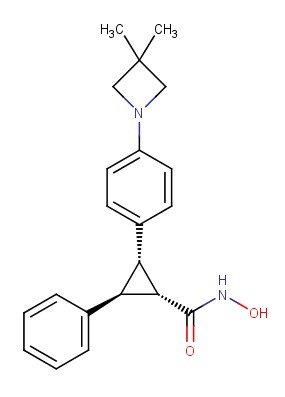 | | 6.48 | 7.16 |
| 2. | S2* | 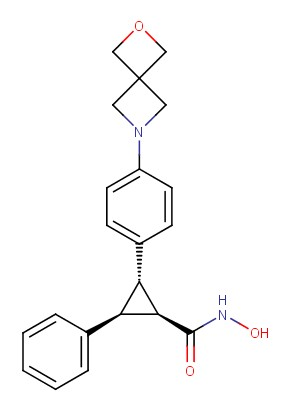 | | 6.82 | 6.80 |
| 3. | S3 | 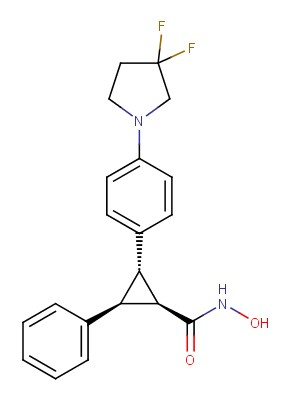 | | 6.82 | 6.99 |
| 4. | S4 | 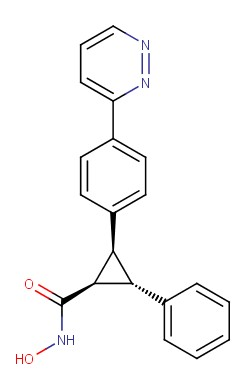 | | 7.09 | 7.20 |
| 5. | S5 | 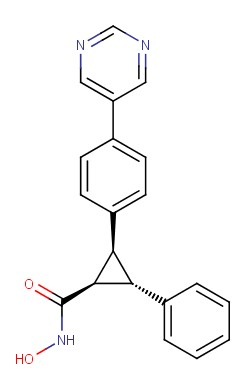 | | 7.00 | 6.88 |
| 6. | S6 | 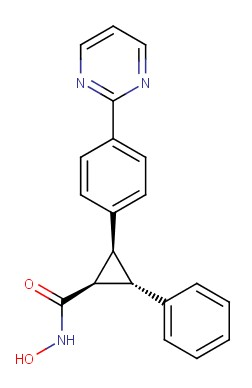 | | 7.22 | 6.93 |
| 7. | S7 | 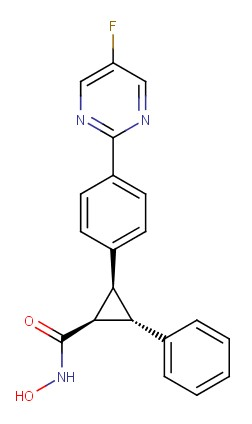 | | 7.30 | 7.12 |
| 8. | S8* | 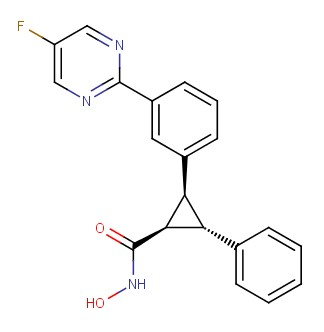 | | 7.00 | 7.34 |
| 9. | S9 | 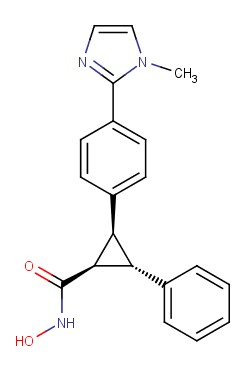 | | 6.53 | 6.81 |
| 10. | S10 | 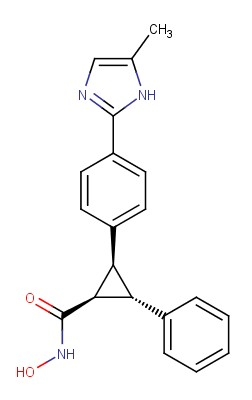 | | 7.00 | 7.11 |
| 11. | S11 | 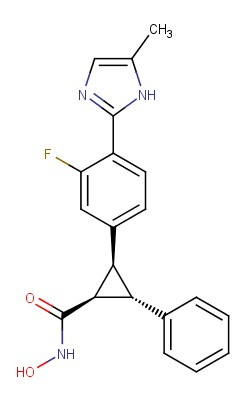 | | 7.22 | 6.78 |
| 12. | S12 | 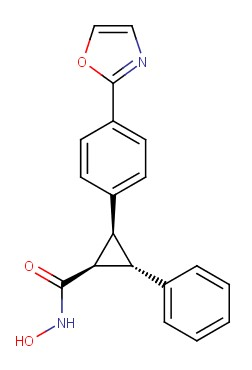 | | 7.30 | 6.92 |
| 13. | S13 | 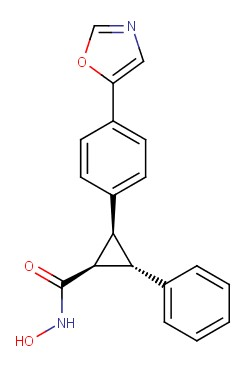 | | 7.69 | 7.37 |
| 14. | S14 | 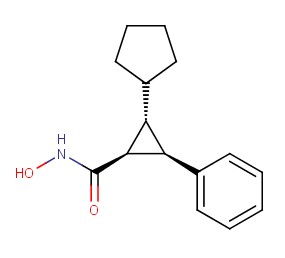 | | 5.04 | 5.12 |
| 15. | S15* | **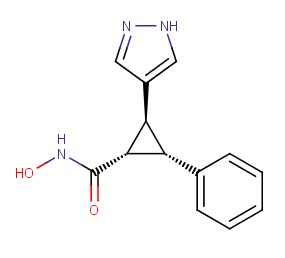** | | 6.26 | 7.05 |
| 16. | S16 | 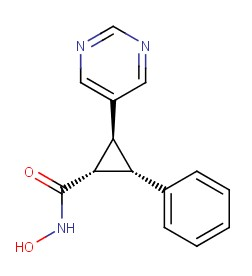 | | 6.27 | 7.04 |
| 17. | S17 | 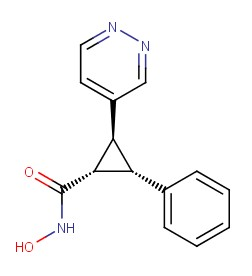 | | 7.00 | 7.06 |
| 18. | S18 | 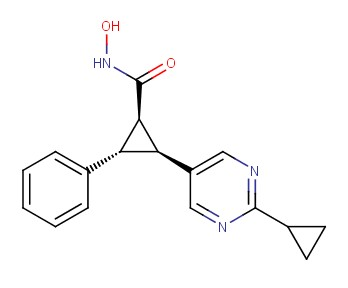 | | 5.74 | 5.96 |
| 19. | S19 | 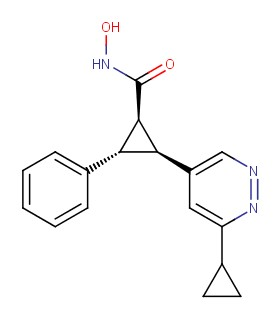 | | 5.95 | 6.74 |
| 20. | S20 | 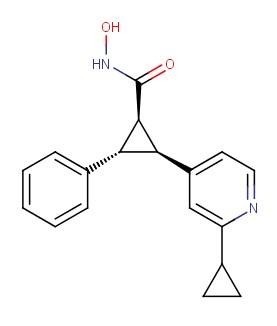 | | 6.30 | 6.20 |
| 21. | S21* | 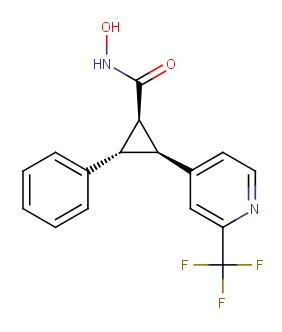 | | 6.55 | 6.40 |
| 22. | S22 | 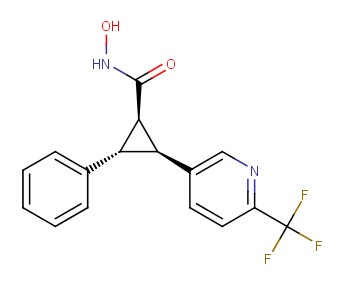 | | 7.69 | 6.61 |
| 23. | S23 | 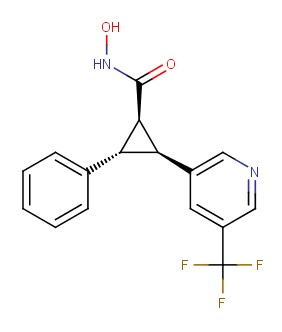 | | 6.52 | 6.55 |
| 24. | S24 | 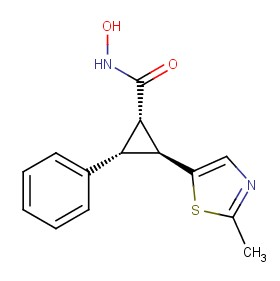 | | 6.46 | 7.08 |
| 25. | S25* | 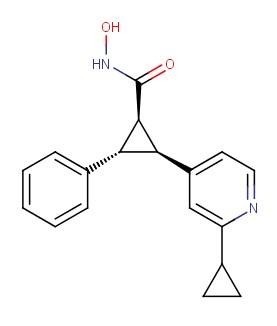 | | 6.63 | 6.20 |
| 26. | S26* | 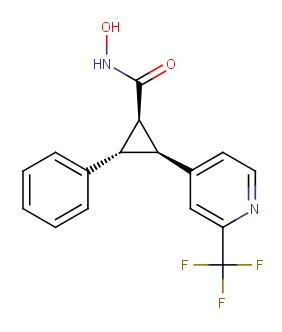 | | 7.00 | 6.40 |
| 27. | S27 | 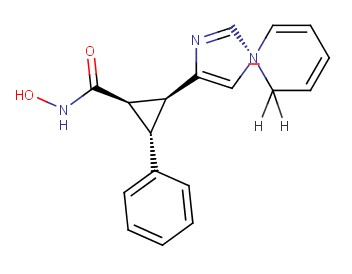 | | 7.69 | 7.06 |
| 28. | S28 | 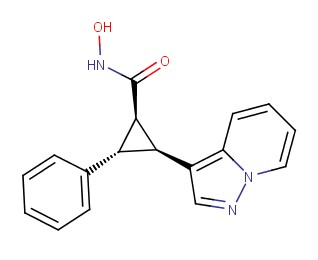 | | 6.52 | 6.74 |
| 29. | S29 | 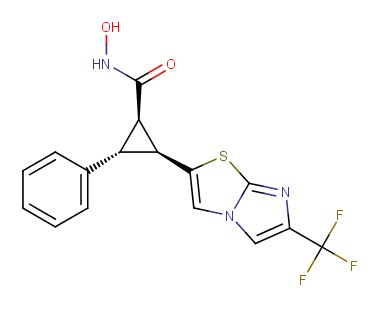 | | 5.82 | 7.29 |
| 30. | S30 | 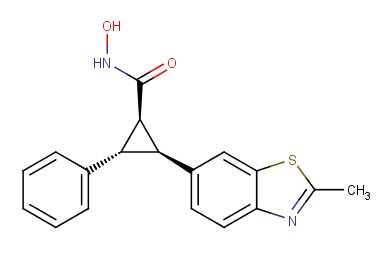 | | 6.17 | 6.99 |
| 31. | S31 | 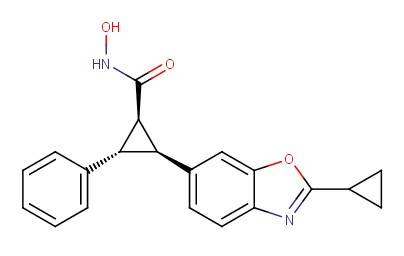 | | 6.69 | 6.88 |
| 32. | S32 | 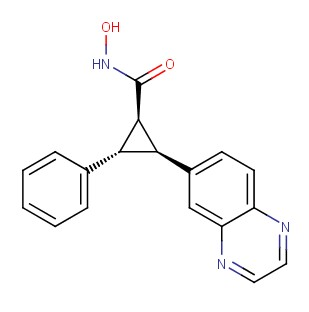 | | 8.00 | 7.41 |
| 33. | S33* | 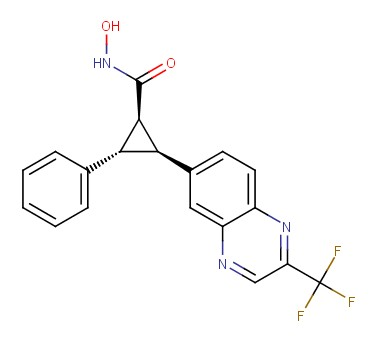 | | 6.84 | 7.42 |
| 34. | S34 | 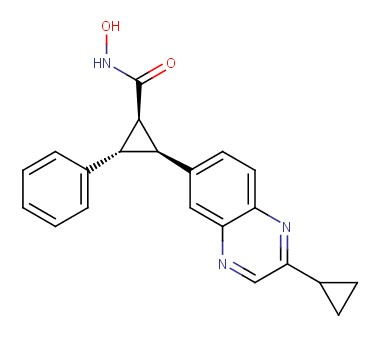 | | 7.69 | 7.53 |
| 35. | S35 | 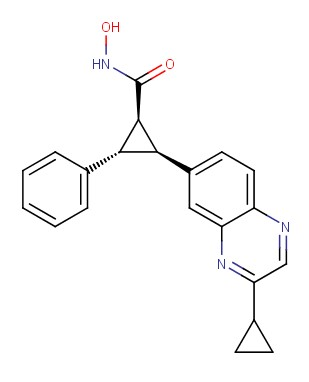 | | 7.09 | 7.34 |
| 36. | S36 | 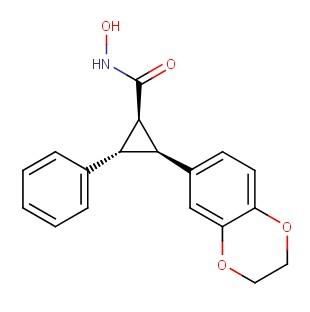 | | 7.69 | 6.98 |
| 37. | S37 | 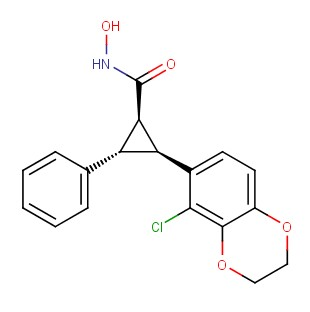 | | 7.52 | 7.00 |
| 38. | S38 | 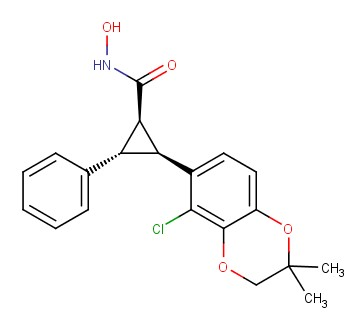 | | 7.52 | 7.03 |
| 39. | S39 | 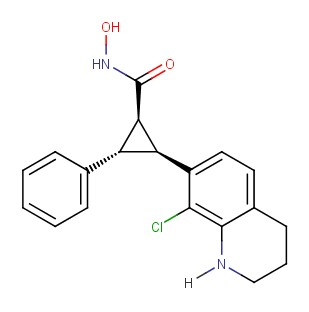 | | 7.69 | 7.15 |
| 40. | S40 | 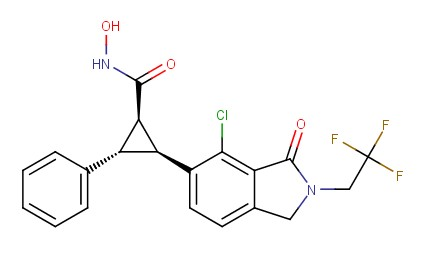 | | 7.39 | 7.46 |
| 41. | S41 | 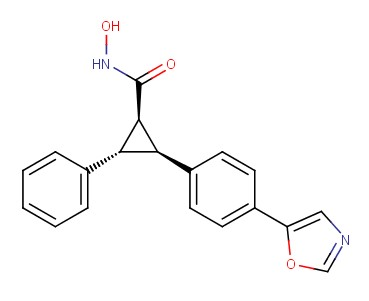 | | 7.39 | 7.12 |
| 42. | S42 | 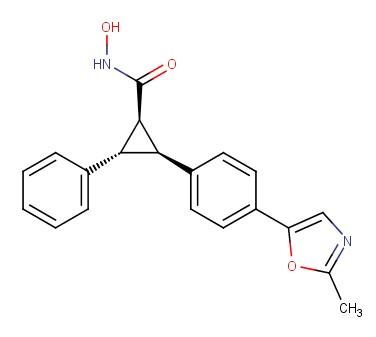 | | 7.52 | 7.15 |
| 43. | S43 | 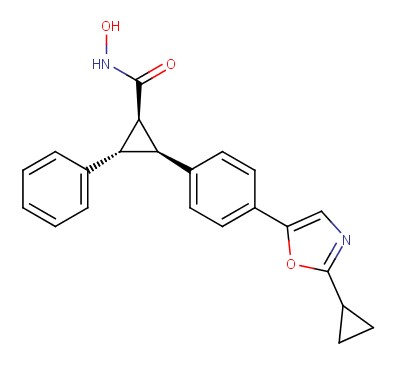 | | 7.69 | 7.33 |
| 44. | S44* |  | | 7.52 | 7.12 |

*test set

**Table S2: Predicted activity of the top scoring compound screened through 3DQSAR model along with the selected descriptors.**

| **S.No** | **Compound ID** | **E_1657** | **S_1911** | **S_1696** | **Predicted Activity** |
| --- | --- | --- | --- | --- | --- |
| **1** | ZINC67902935 | 0.288 | 0.187 | -0.103 | 8.24 |
| **2** | ZINC15063178 | 0.234 | 0.122 | -0.241 | 8.23 |
| **3** | ZINC67902941 | 0.211 | 0.244 | -0.711 | 7.82 |
| **4** | ZINC13373646 | 0.227 | 0.11 | 1.103 | 7.56 |
| **5** | ZINC00608101 | 0.220 | 0.203 | -0.266 | 7.53 |
| **6** | ZINC13386229 | 0.223 | 0.301 | -1.236 | 7.46 |
| **7** | ZINC49823152 | 0.21 | 0.27 | -0.07 | 7.43 |
| **8** | ZINC13311192 | 0.206 | 0.111 | -0.62 | 7.42 |
| **9** | ZINC13311190 | 0.224 | 0.319 | -0.446 | 7.41 |
| **10** | ZINC03881959 | 0.226 | 0.14 | -1.066 | 7.37 |
| **11** | ZINC01996081 | 0.211 | 0.154 | -1.062 | 7.34 |
| **12** | ZINC13374322 | 0.212 | 0.114 | -0.118 | 7.34 |
| **13** | ZINC15043210 | 0.213 | 0.276 | -0.184 | 7.33 |
| **14** | ZINC12153094 | 0.241 | 0.372 | -1.287 | 7.29 |
| **15** | ZINC67902883 | 0.212 | 0.243 | -0.023 | 7.28 |
| **16** | ZINC19702930 | 0.212 | 0.209 | -0.206 | 7.22 |
| **17** | ZINC15063181 | 0.22 | 0.307 | -0.451 | 7.22 |
| **18** | ZINC00898098 | 0.273 | 0.126 | -0.31 | 7.2 |
| **19** | ZINC00387871 | 0.236 | 0.103 | -0.17 | 7.16 |
| **20** | ZINC13380829 | 0.226 | 0.471 | -0.76 | 7.15 |
| **21** | ZINC14966421 | 0.247 | 0.124 | -0.874 | 7.15 |
| **22** | ZINC01996080 | 0.243 | 0.12 | -0.11 | 7.12 |
| **23** | ZINC13386230 | 0.213 | 0.104 | -1.254 | 7.12 |
| **24** | ZINC15043216 | 0.23 | 0.12 | -0.364 | 7.12 |
| **25** | ZINC03870412 | 0.231 | 0.11 | -0.233 | 7.11 |
| **26** | ZINC05022623 | 0.244 | 0.207 | -1.461 | 7.08 |
| **27** | ZINC03943903 | 0.21 | 0.26 | -0.11 | 7.06 |
| **27** | ZINC12153092 | 0.21 | 0.16 | -1.741 | 7.06 |
| **28** | ZINC03881962 | 0.211 | 0.271 | -1.07 | 7.05 |
| **30** | ZINC03918304 | 0.23 | 0.102 | -1.702 | 7.04 |
| **31** | ZINC13374325 | 0.232 | 0.113 | -0.451 | 7.03 |
| **32** | ZINC15249199 | 0.216 | 0.267 | -1.006 | 7.02 |
| **33** | ZINC13374324 | 0.203 | 0.202 | -0.127 | 7 |
| **34** | ZINC00477939 | 0.243 | 0.36 | -0.21 | 6.99 |
| **30** | ZINC12153095 | 0.227 | 0.117 | -0.104 | 6.99 |
| **36** | ZINC13374323 | 0.222 | 0.111 | -0.451 | 6.98 |
| **37** | ZINC20464210 | 0.231 | 0.127 | -0.101 | 6.94 |
| **38** | ZINC01668769 | 0.24 | 0.121 | -0.003 | 6.93 |
| **39** | ZINC03875408 | 0.246 | 0.266 | -1.468 | 6.92 |
| **40** | ZINC01568212 | 0.23 | 0.102 | -0.14 | 6.88 |
| **41** | ZINC12419084 | 0.226 | 0.1 | -0.6 | 6.88 |
| **42** | ZINC00329110 | 0.213 | 0.124 | -0.108 | 6.84 |
| **43** | ZINC00897385 | 0.22 | 0.27 | -0.216 | 6.82 |
| **44** | ZINC20465875 | 0.212 | 0.107 | -0.763 | 6.81 |
| **45** | ZINC02561150 | 0.224 | 0.26 | -0.633 | 6.81 |
| **46** | ZINC00477938 | 0.202 | 0.204 | -0.01 | 6.8 |
| **47** | ZINC03871640 | 0.212 | 0.2 | -0.241 | 6.78 |
| **48** | ZINC03978503 | 0.21 | 0.123 | -0.621 | 6.74 |
| **49** | ZINC12153093 | 0.22 | 0.11 | -0.451 | 6.74 |
| **50** | ZINC15112225 | 0.236 | 0.107 | -0.652 | 6.74 |
| **51** | ZINC04654801 | 0.22 | 0.271 | 0.112 | 6.61 |
| **52** | ZINC04654812 | 0.301 | 0.247 | -1.601 | 6.55 |
| **53** | ZINC67902505 | 0.2 | 0.2 | -0.103 | 6.53 |
| **54** | ZINC15249191 | 0.213 | 0.131 | -0.852 | 6.46 |
| **55** | ZINC67902500 | 0.207 | 0.12 | -0.703 | 6.42 |
| **56** | ZINC67902495 | 0.212 | 0.137 | -0.265 | 6.41 |
| **57** | ZINC03979627 | 0.21 | 0.208 | -0.307 | 6.4 |
| **58** | ZINC11592982 | 0.231 | 0.246 | -0.829 | 6.4 |
| **59** | ZINC67902974 | 0.2 | 0.27 | -0.003 | 6.39 |
| **60** | ZINC15249204 | 0.21 | 0.23 | -0.1 | 6.37 |
| **61** | ZINC59727747 | 0.212 | 0.111 | -0.2 | 6.37 |
| **62** | ZINC49888963 | 0.216 | 0.242 | -0.841 | 6.34 |
| **63** | ZINC67902568 | 0.21 | 0.24 | -0.204 | 6.34 |
| **64** | ZINC67902880 | 0.2 | 0.271 | -0.621 | 6.33 |
| **65** | ZINC67902477 | 0.21 | 0.207 | -1.054 | 6.29 |
| **66** | ZINC03978800 | 0.23 | 0.104 | -0.31 | 6.2 |
| **67** | ZINC05022629 | 0.216 | 0.3 | -0.11 | 6.2 |
| **68** | ZINC15919781 | 0.213 | 3.16 | -0.11 | 6.2 |
| **69** | ZINC15259622 | 0.224 | 0.202 | -1.026 | 6.16 |
| **70** | ZINC67902879 | 0.211 | 0.207 | -1.037 | 6.15 |
| **71** | ZINC67902469 | 0.21 | 0.11 | -0.221 | 6.14 |
| **72** | ZINC49823152 | 0.216 | 0.204 | -0.442 | 6.12 |
| **73** | ZINC59729562 | 0.207 | 0.132 | -0.003 | 6.12 |
| **74** | ZINC59149219 | 0.2 | 0.267 | -0.221 | 6.11 |
| **75** | ZINC00518218 | 0.212 | 0.1 | -0.245 | 6.08 |
| **76** | ZINC15219735 | 0.237 | 0.1 | -0.231 | 6.08 |
| **77** | ZINC67902876 | 0.213 | 0.202 | -0.74 | 6.08 |
| **78** | ZINC67902471 | 0.21 | 0.201 | 0.124 | 6.06 |
| **79** | ZINC59729618 | 0.2 | 0.272 | -0.216 | 6.05 |
| **80** | ZINC15219735 | 0.23 | 0.14 | -1.47 | 6 |
| **81** | ZINC67902860 | 0.2 | 0.47 | -0.23 | 6 |
| **82** | ZINC15657732 | 0.214 | 0.144 | -0.203 | 5.99 |
| **83** | ZINC15216593 | 0.212 | 0.173 | -0.34 | 5.98 |
| **84** | ZINC67902744 | 0.21 | 0.203 | -0.168 | 5.98 |
| **85** | ZINC15259619 | 0.226 | 0.2 | -0.13 | 5.97 |
| **86** | ZINC03973253 | 0.213 | 0.226 | -0.81 | 5.96 |
| **87** | ZINC17654711 | 0.227 | 0.103 | -0.021 | 5.93 |
| **88** | ZINC59587489 | 0.236 | 0.1 | -0.737 | 5.92 |
| **89** | ZINC15249194 | 0.213 | 0.1 | -0.333 | 5.88 |
| **90** | ZINC56874795 | 0.26 | 0.112 | -0.451 | 5.81 |
| **91** | ZINC15259626 | 0.231 | 0.123 | -0.451 | 5.8 |
| **92** | ZINC59402085 | 0.211 | 0.2 | -0.244 | 5.78 |
| **93** | ZINC67902474 | 0.223 | 0.2 | -0.304 | 5.74 |
| **94** | ZINC17021318 | 0.23 | 0.202 | -1.006 | 5.71 |
| **95** | ZINC15063184 | 0.236 | 0.207 | -0.541 | 5.54 |
| **96** | ZINC67903017 | 0.211 | 0.207 | -0.34 | 5.51 |
| **97** | ZINC67902490 | 0.22 | 0.206 | -0.367 | 5.37 |
| **98** | ZINC15063187 | 0.21 | 0.2 | -0.365 | 5.28 |
| **99** | ZINC15259616 | 0.211 | 0.107 | -0.76 | 5.21 |
| **100** | ZINC03881960 | 0.26 | 0.2 | -0.74 | 5.12 |

**Table S3: The ADME properties for SEI and ACI**

| S.no | Molecular property | SEI | ACI |
| --- | --- | --- | --- |
| 1. | #stars | 0 | 3 |
| 2. | #amine | 0 | 0 |
| 3. | #amidine | 0 | 0 |
| 4. | #acid | 0 | 0 |
| 5. | #amide | 0 | 0 |
| 6. | #rotor | 0 | 6 |
| 7. | #rtvFG | 0 | 0 |
| 8. | CNS | 0 | 2 |
| 9. | mol_MW | 391.742 | 305.953 |
| 10 | dipole | 5.652 | 5.518 |
| 11 | SASA | 593.834 | 505.785 |
| 12 | FOSA | 85.337 | 84.979 |
| 13 | FISA | 34.797 | 33.077 |
| 14 | PISA | 263.423 | 236.231 |
| 15 | WPSA | 210.278 | 148.498 |
| 16 | volume | 1068.979 | 1021.571 |
| 17 | donorHB | 3 | 3 |
| 18 | accptHB | 1.7 | 1.2 |
| 19 | dip^2/V | 0.029881 | 0.028483 |
| 20 | ACxDN^.5/SA | 0.04958 | 0.04861 |
| 21 | glob | 0.851424 | 0.836496 |
| 22 | QPpolrz | 35.29 | 35.174 |
| 23 | QPlogPC16 | 12.431 | 11.515 |
| 24 | QPlogPw | 7.995 | 7.854 |
| 25 | QPlogPo/w | 5.631 | 4.762 |
| 26 | QPlogS | -5.811 | -6.194 |
| 27 | CIQPlogS | -6.934 | -6.183 |
| 28 | QPlogHERG | -5.192 | -5.304 |
| 29 | QPPCaco | 4633.655 | 4605.353 |
| 30 | QPlogPoct | 17.834 | 16.897 |
| 31 | QPPMDCK | 10000 | 10000 |
| 32 | QPlogKp | -1.657 | -1.758 |
| 33 | QPlogBB | 0.398 | 0.278 |
| 34 | IP(eV) | 4.643 | 3.802 |
| 35 | #metab | 5 | 5 |
| 36 | QPlogKhsa | 0.672 | 0.578 |
| 37 | Human Oral Absorbtion | 2 | 3 |
| 38 | PercentHumanOralAbsorption | 100 | 100 |
| 39 | SAfluorine | 0 | 0 |
| 40 | RuleOfFive | 1 | 1 |
| 41 | #ringatoms | 16 | 15 |
| 42 | #nonHatm | 23 | 23 |
| 43 | #in34 | 0 | 2 |
| 44 | SAamideO | 0 | 0 |
| 45 | PSA | 24.539 | 22.353 |
| 46 | #NandO | 3 | 3 |
| 47 | RuleOfThree | 1 | 1 |
| 48 | #in56 | 16 | 14 |
| 49 | #noncon | 3 | 2 |
| 50 | EA(eV) | 0.561 | 0.503 |
